# Long noncoding RNA *PM* maintains cerebellar synaptic integrity and *Cbln1* activation via Pax6/Mll1-mediated H3K4me3

**DOI:** 10.1371/journal.pbio.3001297

**Published:** 2021-06-10

**Authors:** Yan Jin, Bowen Zhang, Junxia Lu, Yingdong Song, Wei Wang, Wei Zhang, Fanghong Shao, Meng Gong, Meiting Wang, Xiaolin Liang, Shuqin Li, Zhi Zhang, Ge Shan, Xiangting Wang

**Affiliations:** 1 Department of Geriatrics, Gerontology Institute of Anhui Province, The First Affiliated Hospital, Division of Life Sciences and Medicine, University of Science and Technology of China, Hefei, China; 2 Anhui Provincial Key Laboratory of Tumor Immunotherapy and Nutrition Therapy, Hefei, China; 3 Hefei National Laboratory for Physical Sciences at the Microscale, University of Science and Technology of China, Hefei, China; 4 The Chinese Academy of Sciences Key Laboratory of Innate Immunity and Chronic Disease, School of Basic Medical Sciences, Division of Life Sciences and Medicine, University of Science and Technology of China, Hefei, China; 5 Liren College, Yanshan University, Qinhuangdao, China; 6 Department of Neurobiology and Biophysics, Division of Life Sciences and Medicine, University of Science and Technology of China, Hefei, China; 7 Department of Pathophysiology, Hebei Medical University, Shijiazhuang, China; 8 Department of Anesthesiology, The First Affiliated Hospital, Division of Life Sciences and Medicine, University of Science and Technology of China, Hefei, China; Centro de Investigacion y de Estudios Avanzados del Instituto Politecnico Nacional, MEXICO

## Abstract

Recent studies have shown that long noncoding RNAs (lncRNAs) are critical regulators in the central nervous system (CNS). However, their roles in the cerebellum are currently unclear. In this work, we identified the isoform 204 of lncRNA *Gm2694* (designated as *lncRNA-Promoting Methylation* (*lncRNA-PM*)) is highly expressed in the cerebellum and derived from the antisense strand of the upstream region of *Cerebellin-1* (*Cbln1*), a well-known critical cerebellar synaptic organizer. *LncRNA-PM* exhibits similar spatiotemporal expression pattern as *Cbln1* in the postnatal mouse cerebellum and activates the transcription of *Cbln1* through Pax6/Mll1-mediated H3K4me3. In mouse cerebellum, *lncRNA-PM*, Pax6/Mll1, and H3K4me3 are all associated with the regulatory regions of *Cbln1*. Knockdown of *lncRNA-PM* in cerebellum causes deficiencies in *Cbln1* expression, cerebellar synaptic integrity, and motor function. Together, our work reveals an lncRNA-mediated transcriptional activation of *Cbln1* through Pax6-Mll1-H3K4me3 and provides novel insights of the essential roles of lncRNA in the cerebellum.

## Abstract 2

The long non-coding RNA lncRNA-PM activates transcription of the cerebellar synaptic organizer Cbln1 by promoting Pax6-Mll1-mediated H3K4me3 methylation, thereby helping to maintain cerebellar synaptic integrity and motor function.

## Introduction

Cerebellum plays a crucial role in motor functions including coordination, posture, and balance [1–6]. The cerebellar cortex consists of 3 sagittal-orientated zones: molecular layer, Purkinje cell (PC) layer, and granule cell (GC) layer [1–6]. A core cerebellar circuit, which mediates all of the cerebellar functions, is mainly comprised of GCs and PCs [1,5]. GCs project parallel fibers (PFs) and send excitatory signals to PCs. The growing PFs form thousands of synapses with the dendritic arborization of PCs in the molecular layer. PCs function as the sole output of the cerebellar cortex.

Cerebellin-1 (Cbln1) plays essential roles in cerebellar synaptic integrity and plasticity. It is expressed and secreted from the GCs and helps to establish and maintain the synaptic connections between PFs (axons of the GCs) and the dendrites of PCs through interaction with the presynaptic neurexin (Nrx) and the postsynaptic glutamate receptor delta2 (GluD2) [7–10]. *Cbln1*-null mice exhibited profound reduction in the number of synapses formed between PFs and PCs during development [7]. The observations that Cbln1 could rapidly induce the formation of functional and structurally normal PF synapses in the adult murine cerebellum indicated that Cbln1 functions in the mature neurons as well [11]. However, despite its well-known functions and therapeutic potential for cerebellar ataxic disorders, the gene expression regulation of *Cbln1* is yet to be elucidated.

Long noncoding RNAs (lncRNAs) are enormously enriched in the central nervous system (CNS) [12]. Despite some reports on the role of lncRNAs in the CNS [13–15], how lncRNAs are involved in the brain function and regulation is largely unexplored. Recently, we and other independent groups have revealed dynamic spatiotemporal expression patterns of lncRNAs in mammalian brain [16–19]. Interestingly, we found that the number of cerebellar-expressing lncRNAs are relatively higher and that their profiling is quite unique compared with the other tested brain areas [16,17], raising a possibility that these cerebellum highly expressed lncRNAs may be essential regulators of cerebellar function.

In the present study, we found that our designated *lncRNA-PM* (*Gm2694-204*) is a specific isoform of *Gm2694* to promote the expression of *Cbln1*. *LncRNA-PM* increases the DNA-bound fractions of Pax6 and Mll1, promotes the recruitment of Pax6, Mll1, and H3K4me3 to the upstream regulatory regions of *Cbln1*, and thus activates *Cbln1* transcription in cultured Neuro2a cells. In the mouse cerebellum, *lncRNA-PM*, Pax6, and Mll1 are associated and located to the high H3K4me3 marked regions of *Cbln1*. Knockdown of *lncRNA-PM* specifically in the cerebellum causes deficiencies in *Cbln1* expression, cerebellar synaptic integrity, and motor function. Moreover, we found that *Gm2694* has a broader distribution in the cerebellum than *Cbln*1 through data mining of the reported cerebellar single-cell data and that *lncRNA-PM* regulates genes functioning in GABAergic synaptic transmission and morphogenesis of branching structure. Collectively, we characterize *lncRNA-PM* as a regulator of *Cbln1* transcriptional activation and suggest *lncRNA-PM* as a multifunctional regulator in the cerebellum.

## Results

### Identification of *lncRNA-PM*, a splicing isoform of *Gm2694*, which promotes *Cbln1* transcriptional activation

**Fig 1:**
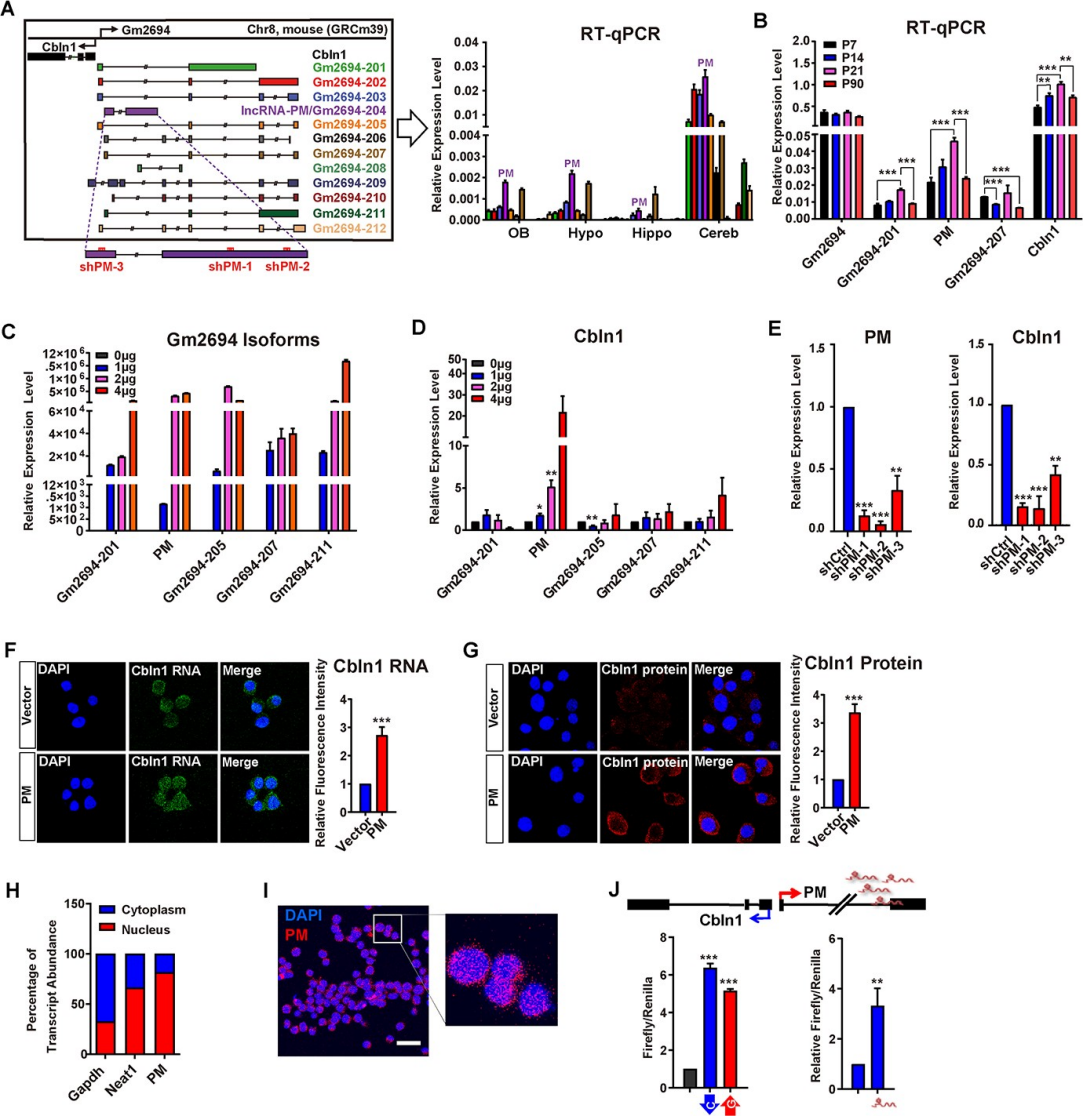
Identification of *lncRNA-PM*, a splicing isoform of *Gm2694*, which is required for *Cbln1* activation. (A) Left: illustration of 12 *Gm2694* isoforms indicated as different colors. The sh*PM*-1, sh*PM*-2, and sh*PM*-3 used in the study were indicated. Right: the relative expression levels of the 12 isoforms of *Gm2694* in the indicated brain regions. Data are shown as means ± SEMs, *n =* 3. *Gm2694-204* is designated as *lncRNA-PM* (PM). (B) The relative expression levels of *Gm2694* (for *201*, *202*, *203*, *205*, *206*, *207*, *209*, and *210*), *Gm2694-201*, *PM*, *Gm2694-205*, *Gm2694-207*, and *Cbln1* at the indicated cerebellar developmental time points were measured by RT-qPCR. Data are shown as means ± SEMs, *n =* 3. (C, D) The expression levels of the indicated *Gm2694* isoforms (C) and *Cbln1* (D) under the indicated treatments. (E) The expression levels of *Cbln1* in control or *PM* shRNAs (sh*PM*-1/2/3)-treated Neuro2a cells, detected by RT-qPCR. Data are shown as means ± SEMs, *n =* 3. (F) Left: representative images of *Cbln1* mRNA in the control or *PM*-overexpressed Neuro2a cells, detected by FISH. Right: quantification of the left. Data are shown as means ± SEMs, *n =* 3. (G) Left: representative immunofluorescence images of *Cbln1* protein in the control (Vector) or *PM-*overexpressed (PM) Neuro2a cells. Right: quantification of the left. Data are shown as means ± SEMs, *n =* 3. (H) Subcellular distributions of *PM*, *Gapdh*, and *Neat1* by fractionating assay in Neuro2a cells. *Gapdh* and *Neat1* RNA served as positive controls for RNAs predominantly expressed in cytoplasm and nucleus, respectively. Data are shown as means ± SEMs, *n* = 3. (I) Representative FISH image of *PM* RNA (red) in Neuro2a cells. Nuclei were stained with DAPI (blue). (J) Top: schematic illustration of the *Cbln1*/*PM* locus. Bottom left: luciferase assays indicate the activity changes of pGL3-*Cbln1* (blue) and pGL3*-Gm2694* (red), compared with pGL3 vector control (black). Bottom right: Luciferase assays indicate the activity changes of pGL3-*Cbln1* in the presence of control or *PM*-expressing plasmid. Data are shown as means ± SEMs, *n =* 3. Scale bar, 13 um. All RT-qPCR results were normalized to *Gapdh*. All the data of this figure can be found in the S1 Data file. *P < 0.05, **P < 0.01, and ***P < 0.001. *Cbln1*, *Cerebellin-1*; Cereb, Cerebellum; FISH, fluorescence in situ hybridization; Hippo, hippocampus; Hypo, hypothalamus; *lncRNA-PM*, *lncRNA-Promoting Methylation*; OB, olfactory bulb; RT-qPCR, reverse transcription-quantitative PCR.

Our previous work showed that cerebellum is enriched for lncRNAs [17]. In order to explore the biological roles of lncRNAs in the cerebellum, we further analyzed the 57 identified cerebellum highly expressed lncRNAs (representing 63 reported transcripts) (S1 Table; [17]). Among these, lncRNA *Gm2694* is derived from the antisense strand of the upstream region of *Cbln1* (Fig 1A, left panel), exhibiting as a divergent lncRNA gene to *Cbln1*. Previous study has reported that “divergent lncRNAs” often positively regulate the transcription of their nearby coding genes and participate in similar biological processes as the neighboring genes [20]. Through reverse transcription-quantitative PCR (RT-qPCR), our results showed that lncRNA *Gm2694* is relatively highly expressed in the cerebellum than olfactory bulb, hypothalamus, and hippocampus (Fig 1A, right panel). Such relatively high cerebellar expression of *Gm2694* is similar to *Cbln1* [7,21]. Moreover, data mining from the available developing cerebellum single-cell RNA sequencing (RNA-seq) datasets also revealed that *Gm2694* and *Cbln1* were coexpressed in certain subgroups of the cerebellar GCs and cerebellar nuclear neurons (S1 Fig; [21,22]). These results raised a very likely possibility that *Gm2694* acts as a regulator of *Cbln1* in these cells. Owing to the known essential role of Cbln1 as a synaptic organizer in the cerebellum and the unknown regulatory mechanisms of its expression, we focused our attention on the *Cbln1* neighboring lncRNA, *Gm2694*, in this study.

The Ensembl database (version 101) has cataloged 12 splicing isoforms of *Gm2694* (Fig 1A, left panel). Our qPCR results showed that except for isoforms 208 and 209—which were not detectable in the tested brain samples—the remaining 10 isoforms of *Gm2694* were expressed at higher levels in the postnatal mouse cerebellum than in other tested brain regions (Fig 1A, right panel). When we examined cerebellum tissues at 4 key postnatal stages (P7, P14, P21, and P90) [2,4–6], we found that *Gm2694-201*, *Gm2694-204* (*PM*), and *Cbln1* were induced from P7 to P21, and then reduced at P90 (Fig 1B). The net expression of *Gm2694-201*, *202*, *203*, *205*, *206*, *207*, *209*, and *210*, shown as *Gm2694* in Fig 1B, showed to be relatively stable across the 4 tested time points. Next, we cloned 5 abundant *Gm2694* isoforms (*201*, *204*, *205*, *207*, and *211*; Fig 1A, right panel), and tested their gain of functions on *Cbln1* in cultured Neuro2a cells. Surprisingly, despite the similar cerebellum expression patterns among these tested isoforms, only the overexpression of *Gm2694-204* altered *Cbln1* expression and caused a significant increase of *Cbln1* mRNA level (Fig 1C and 1D). Owing to the later identified role of *Gm2694-204* in recruiting H3K4me3 to the regulatory regions of *Cbln1*, we designated *Gm2694-204* as *lncRNA*-*Promoting Methylation* (*PM*). *LncRNA*-*PM* is then used in the following context and all the figures. In order to characterize the 5′ and 3′ of *lncRNA-PM* and *Cbln1* transcripts, we conducted rapid amplification of cDNA ends (RACE) from mouse cerebellum total RNA and confirmed that *lncRNA-PM* and *Cbln1* are positioned head-to-head (S2 Fig). The positive effect of *lncRNA-PM* on *Cbln1* mRNA was further confirmed by RT-qPCR in *lncRNA-PM* knockdown cells (Fig 1E) and by fluorescence in situ hybridization (FISH) analysis in *lncRNA-PM*–overexpressed cells (Fig 1F). Consistently, the protein level of *Cbln1* was dramatically increased in *lncRNA-PM*–overexpressed cells detected by immunofluorescence (Fig 1G). Together, these results indicated that *lncRNA-PM* can promote the transcriptional activation of *Cbln1*.

**Fig 2:**
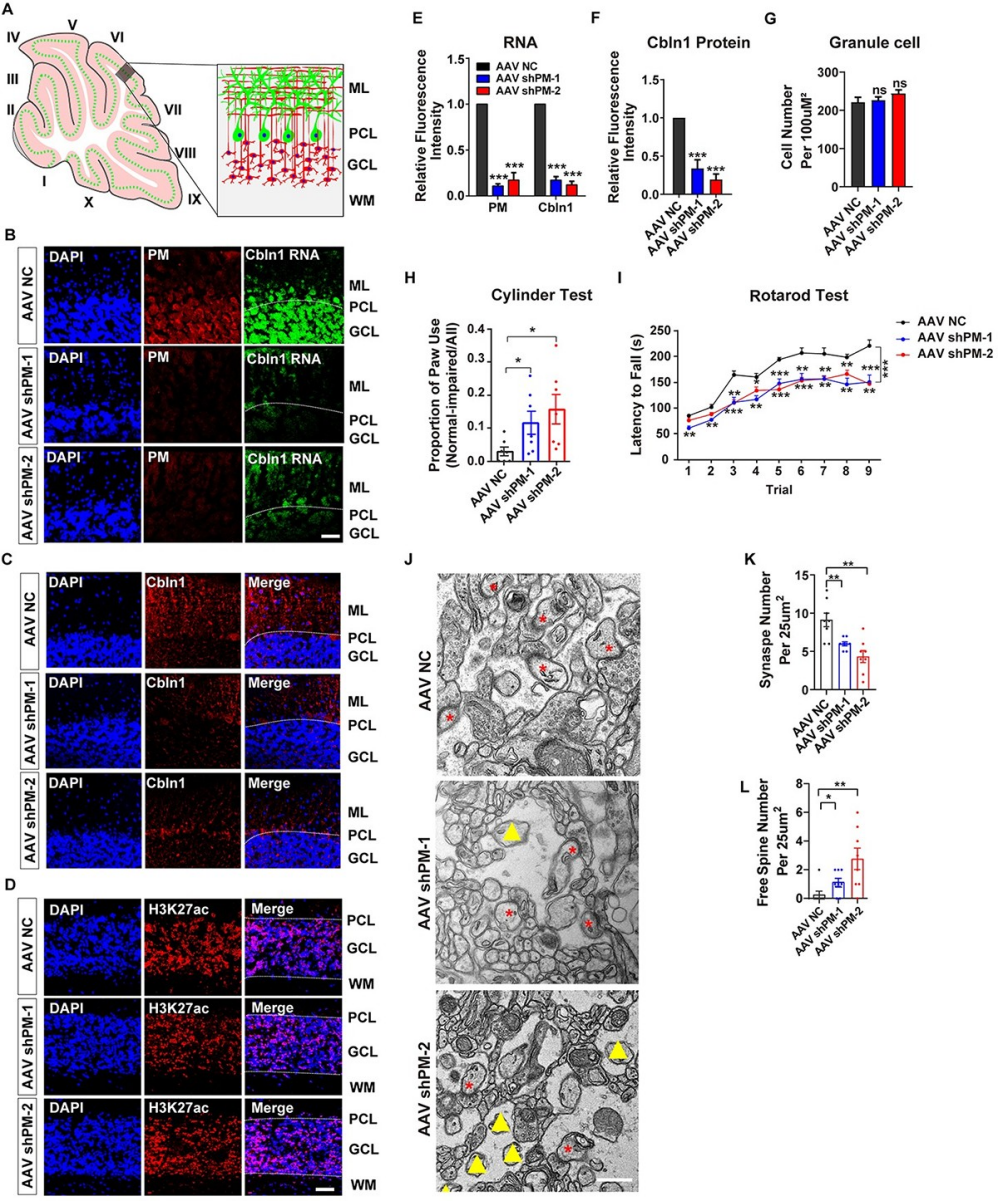
*LncRNA-PM* knockdown in mouse cerebellum results in decreased *Cbln1*, impaired synapse integrity, and motor behavior. (A) Illustrated structure in the investigated cerebellar area. (B) The expression levels of *PM* (red) and *Cbln1* (green) RNA in the cerebellar sections obtained from control (AAV NC) or *lncRNA-PM* (*PM*, red) shRNA-injected mice (AAV sh*PM*-1 and AAV sh*PM*-2). Scale bar, 40 um. (C and D) Immunofluorescence images of *Cbln1* protein (C) and H3K27ac (D) in the cerebellar sections obtained from AAV NC and AAV sh*PM*s mice. Scale bar, 40 um. (E) Quantification of the RNA levels of *PM* and *Cbln1* obtained from B. Data are shown as means ± SEMs, *n =* 3. (F) Quantification of the protein levels of *Cbln1* obtained from C. (G) Quantification of the GC numbers. (H) Evaluation of the control and sh*PM*s-injected mice on cylinder test. Cylinder test was designed to assess limb preference on mice. It showed that compared with the control mice, *PM* shRNA-injected mice exhibited asymmetric high frequency in forepaw usage. The paw usage was counted and analyzed by normal-impaired/all. Data are shown as means ± SEMs, *n =* 7 (per group). (I) Evaluation of the control and sh*PM*s-injected mice on rotarod test. Latency to fall provides the quantification of motor ability on the accelerating rotarod. It showed that compared with the control mice, sh*PM*s-injected mice exhibited significant reduced duration in latency to fall. Data are shown as means ± SEMs, *n* = 6 (per group). (J) Representative electron microscope images for the control and sh*PM*-injected mice. Red asterisk: intact synapse. Yellow triangle: free spine. Scale bar, 500 nm. (K) Quantification of the numbers of intact synapses obtained from J in the indicated treatments. (L) Quantification of the numbers of free spines obtained from (J) in the indicated treatments. All the data of this figure can be found in the S1 Data file. *P < 0.05, **P < 0.01, and ***P < 0.001. *Cbln1*, *Cerebellin-1*; GCL, granule cell layer; *lncRNA-PM*, *lncRNA-Promoting Methylation*; ML, molecular layer; PCL, Purkinje cell layer; WM, white matter.

Next, both fractionation and FISH analysis detected the nuclear expression of *lncRNA-PM* in the cultured Neuro2a cells (Fig 1H and 1I) and the cerebellar GCs (Fig 2B). In order to test whether the effect of *lncRNA-PM* on *Cbln1* is at the transcriptional level, we constructed a pGL3 vector containing the promoter sequence of *Cbln1* (pGL3-*Cbln1*) and observed that overexpression of *lncRNA-PM* could further activate the pGL3-*Cbln1* promoter activity in Neuro2a cells by luciferase assays (Fig 1J). To test the impacts of *lncRNA-PM* on other genes located near the *Cbln1/Gm2694* locus, we designed specific qPCR primer sets for 12 nearby genes, including *4933402J07Rik*, *Gm24841*, *Zfp423*, *Cnep1r1*, *Heatr3*, *Tent4b*, *Brd7*, *N4bp1*, *Siah1a*, *Lonp2*, *Abcc1*2, and *Phkb*. With one exception (*Abcc12*, a relatively distal gene to the *Cbln1/Gm2694* locus), none of these genes displayed significantly altered expression upon *lncRNA-PM* knockdown (S3 Fig). Collectively, our results suggested that *lncRNA-PM* promotes the expression of its divergent gene *Cbln1* at the transcriptional level.

### Selective knockdown of *lncRNA-PM* in mouse cerebellum results in synaptic terminal reduction and motor behavioral deficiencies

Next, we attempted to investigate the in vivo function of *lncRNA-PM*. In order to avoid the removal of DNA *cis*-acting elements on the *Cbln1*/*Gm2694* locus, we chose a knockdown strategy, instead of knockout, for this particular case of *lncRNA-PM*. Control shRNA (negative control, NC) and 2 shRNAs to *lncRNA-PM* exon2 (shPM-1 and shPM-2) (indicated in Fig 1A, left panel) were generated into the pAAV-eGFP backbone and were utilized in the cerebellum injection. The virus infection resulted in wide distribution in the mice cerebellum (S4 Fig). When compared with the negative control, the expression levels of *lncRNA-PM* in the cerebellar GCs were sharply reduced in shPM-injected mice (Fig 2A and 2B). Consistently with the positive regulation of *lncRNA-PM* on *Cbln1* in the Neuro2a cells shown in Fig 1, *lncRNA-PM* knockdown also resulted in sharply reduced expression levels of *Cbln1* mRNA (Fig 2B and 2E) and protein (Fig 2C and 2F) in the shPM-injected mice. In contrast, a control antibody of H3K27ac displayed no difference between negative control and shPM mice (Fig 2D). Note that the number of GCs in the shPM-injected mice showed no significant difference with the negative control-injected mice, eliminating the possibility that the decrease of *Cbln1* could have resulted from an altered number of GCs (Fig 2G).

The rotarod test and cylinder test were designed to assess motor coordination and limb preference on mice. In cylinder test, the shPM-injected mice showed significant asymmetric high frequency of forepaw usage when compared with negative control-injected mice (Fig 2H). In rotarod test, latency to fall provided the quantification of motor ability on the accelerating rotarod, and our data showed a significant decrease of latency to fall in the shPM-injected mice than negative control-injected mice (Fig 2I).

To further confirm the reported PF–PC synapse deficiencies in *Cbln1*-null mice [7], we used electron microscopy to examine the ultrastructure and analysis for the shPM-injected mice. We found that the number of synapses was markedly reduced in the shPM-injected mouse cerebellum, when compared to the negative control-injected mice (Fig 2J and 2K). The “naked” synapses contain postsynaptic density (PSD)-like condensations but lack of presynaptic contact. In the shPM-injected mouse cerebellum, we also observed significantly increased number of “naked” spines (Fig 2L).

**Fig 3:**
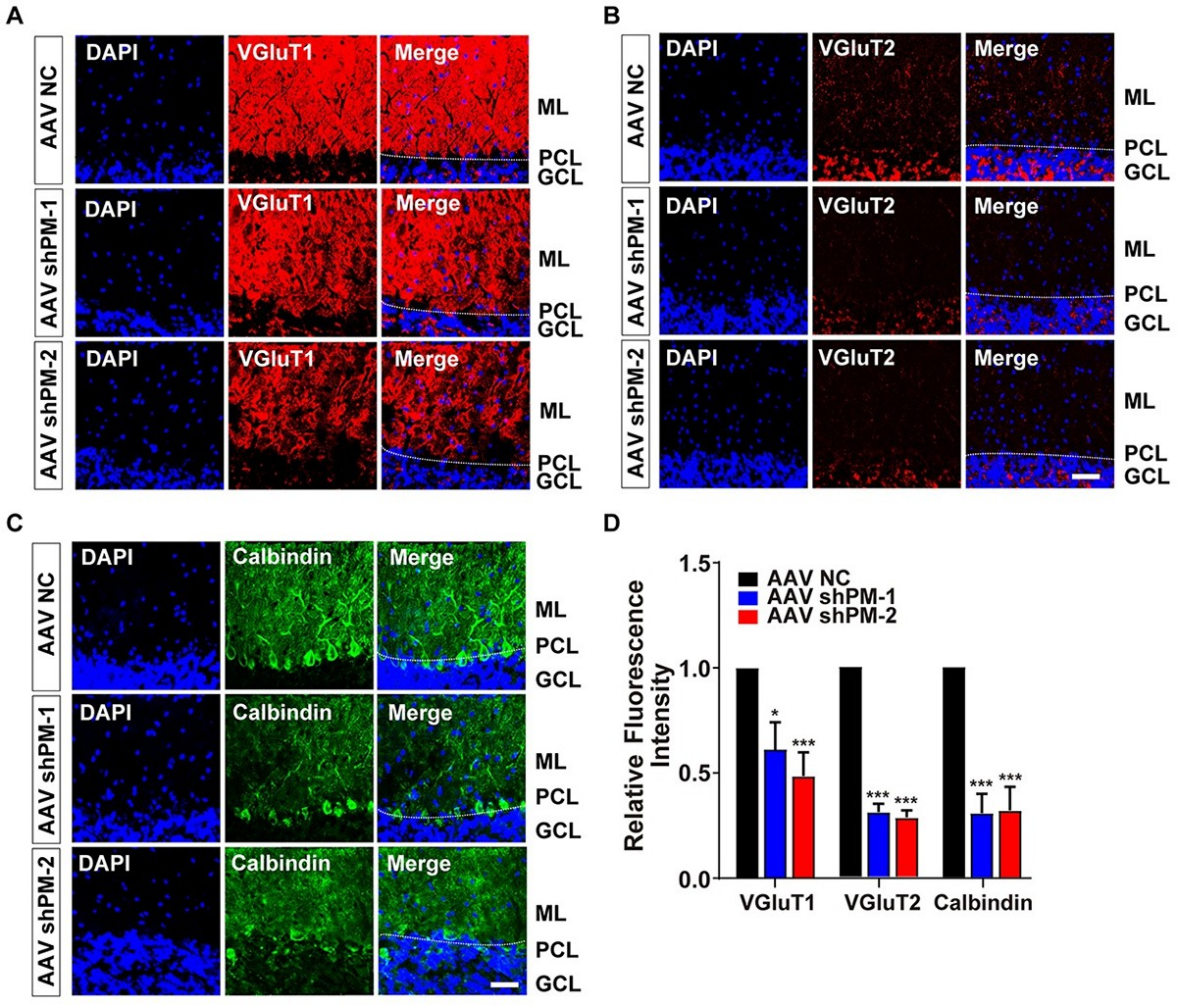
Reduced density of VGluT1, VGluT2, and Calbindin in the shPMs-injected mice. (A–C) Representative immunofluorescence images showed the density of VGluT1 (A), VGluT2 (B), or Calbindin (C) in the control and indicated sh*PM*s-injected mice. VGluT1, VGluT2, and Calbindin are markers for PF terminals, climbing fiber terminals, and PCs, respectively. (D) Quantification of the indicated markers obtained from A–C. All the data of this figure can be found in the S1 Data file. Data are shown as means ± SEMs, *n =* 3. Scale bar, 40 um. *P < 0.05 and ***P < 0.001. GCL, granule cell layer; ML, molecular layer; PCL, Purkinje cell layer; PF, parallel fiber; VGluT1, vesicular glutamate transporter 1; VGluT2, vesicular glutamate transporter 2.

Vesicular glutamate transporter 1 (VGluT1) and VGluT2 are PF and climbing fiber terminals markers, respectively [7]. It is known that *Cbln1*-null mice displayed impaired VGluT1 and VGluT2 expression patterns [7]. In order to examine whether *lncRNA-PM* influenced the general properties of VGluT1 and VGluT2, we performed immunofluorescence assay. Our data showed a significant reduction of VGluT1 (Fig 3A and 3D) and VGluT2 (Fig 3B and 3D) in shPM-injected mice. Moreover, we found that the longitudinal outgrowth of PC dendrites, detected by Calbindin, was decreased in shPM-injected mice (Fig 3C and 3D). Together, our results showed that *lncRNA-PM* is critical for normal synaptic terminal formation and cerebellar motor functions.

### *LncRNA-PM* mediates H3K4me3 on *Cbln1* via Mll1

**Fig 4:**
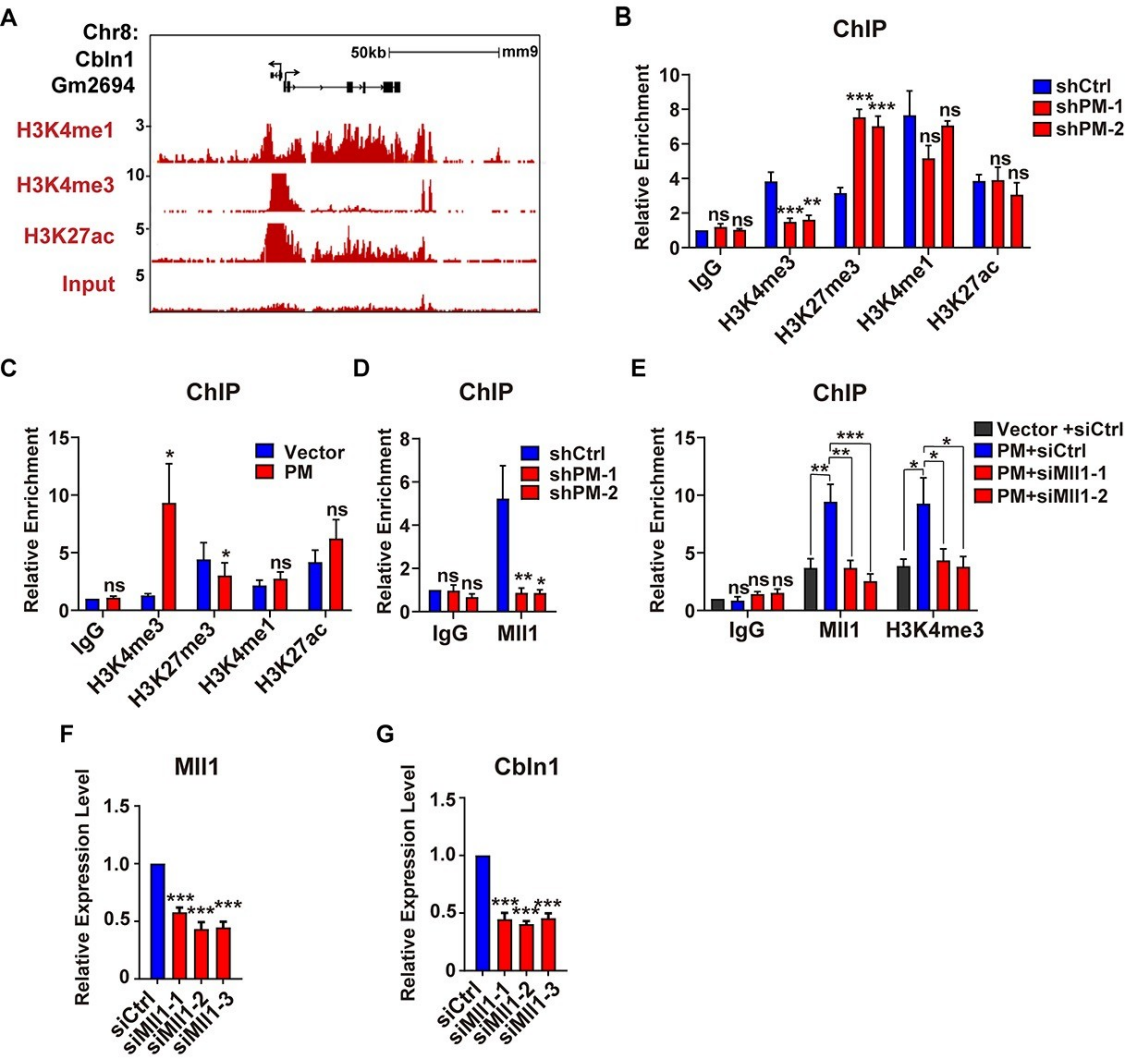
*LncRNA-PM* mediates H3K4me3 recruitment on the 5′ regulatory region of *Cbln1 via* Mll1. (A) Indicated histone markers on *Gm2694/Cbln1* locus in mouse cerebellum based on ENCODE data. (B) ChIP-qPCR detection of the indicated histone markers on the 5′ regulatory region of *Cbln1* in the presence of control (shCtrl) or *PM* shRNAs (sh*PM*-1 and sh*PM*-2) in Neuro2a cells. Data are shown as means ± SEMs, *n =* 3. (C) ChIP-qPCR detection of the indicated histone markers on the 5′ regulatory region of *Cbln1* in the control (Vector) or *PM*-overexpressed (PM) Neuro2a cells. Data are shown as means ± SEMs, *n* = 3. (D) ChIP-qPCR detection of Mll1 on the 5′ regulatory region of *Cbln1* in the control or the indicated sh*PM*s in Neuro2a cells. Data are shown as means ± SEMs, *n* = 3. (E) Changes of the Mll1 and H3K4me3 deposition on the 5′ regulatory region of *Cbln1* in *PM*-overexpressed Neuro2a cells, with the treatments of Mll1 (siMll1-1 and siMll1-2) or control siRNAs (siCtrl). Data are shown as means ± SEMs, *n* = 3. (F and G) The expression levels of *Mll1* (F) and *Cbln1* (G) mRNAs in the control or *Mll1* siRNAs-treated Neuro2a cells. All results were normalized to *Gapdh*. All the data of this figure can be found in the S1 Data file. Data are shown as means ± SEMs, *n* = 3. *P < 0.05, **P < 0.01, and ***P < 0.001. *Cbln1*, *Cerebellin-1*; ChIP, chromatin immunoprecipitation; IgG, immunoglobulin G; *lncRNA-PM*, *lncRNA-Promoting Methylation*; ns, no significance; qPCR, quantitative PCR.

The *Cbln1*-*Gm2694* locus showed highly enrichments of H3K4me3/H3K27ac markers, which encompass the transcription start sites and the upstream regulatory regions of *Cbln1* (Fig 4A; S2 Table). In order to test the effect of *lncRNA-PM* on the histone markers located on the upstream regulatory regions of *Cbln1*, we either knocked down or overexpressed *lncRNA-PM* in cultured Neuro2a cells and performed chromatin immunoprecipitation (ChIP) assays. Our results showed that the H3K4me3 occupancy was consistently and dramatically increased by *lncRNA-PM* (Fig 4B and 4C). In contrast, the H3K27me3 level was consistently significantly decreased by *lncRNA-PM* (Fig 4B and 4C). No significant alteration of H3K4me1 or H3K27ac levels was detected upon modulation of *lncRNA-PM* (Fig 4B and 4C).

Mll family contains 4 members (Mll1-4) and is known for their histone methyltransferase activities. Among them, Mll1 is a specific methyltransferase for H3K4me3 [23,24]. ChIP-qPCR assay showed that overexpression of *lncRNA-PM* in Neuro2a cells increased the recruitment of Mll1 instead of Mll2-Mll4 to the upstream regulatory regions of *Cbln1* (S5 Fig). Consistently, knockdown of *lncRNA-PM* decreased recruitment of Mll1 to the upstream regulatory regions of *Cbln1* (Fig 4D). These results suggested an involvement of Mll1 in *lncRNA-PM*-mediated *Cbln1* activation. To test our hypothesis, we knocked down *Mll1* in *lncRNA-PM*-overexpressed Neuro2a cells and found that the effect of *lncRNA-PM*-induced H3K4me3 enrichment was completely blocked by *Mll1* knockdown (Fig 4E). Supporting these results, *Mll1* knockdown significantly decreased the expression level of *Cbln1* (Fig 4F and 4G). Taken together, our data suggested that *lncRNA-PM*–mediated H3K4me3 of *Cbln1* is Mll1 dependent.

### Pax6 is required for the *lncRNA-PM*-mediated Mll1-H3K4me3 enrichment

It has been reported that Mll1 forms a complex with Pax6 [24], a transcriptional factor that is highly expressed in mouse cerebellum [25] and a potential upstream regulator of *Cbln1* suggested by genome-wide microarray analysis in the developing mouse cerebellum [26]. Very recently, a single-cell transcriptional study of the developing murine cerebellum also identified *Pax6* as a lineage marker for the granule neuron progenitors and granule neurons [22]. Our data mining results showed that cells containing *Gm2694* share strong overlap with *Pax6*-positive cell lineages (S1 Fig). These findings collectively indicated that *lncRNA-PM* may regulate *Cbln1* through Pax6.

**Fig 5:**
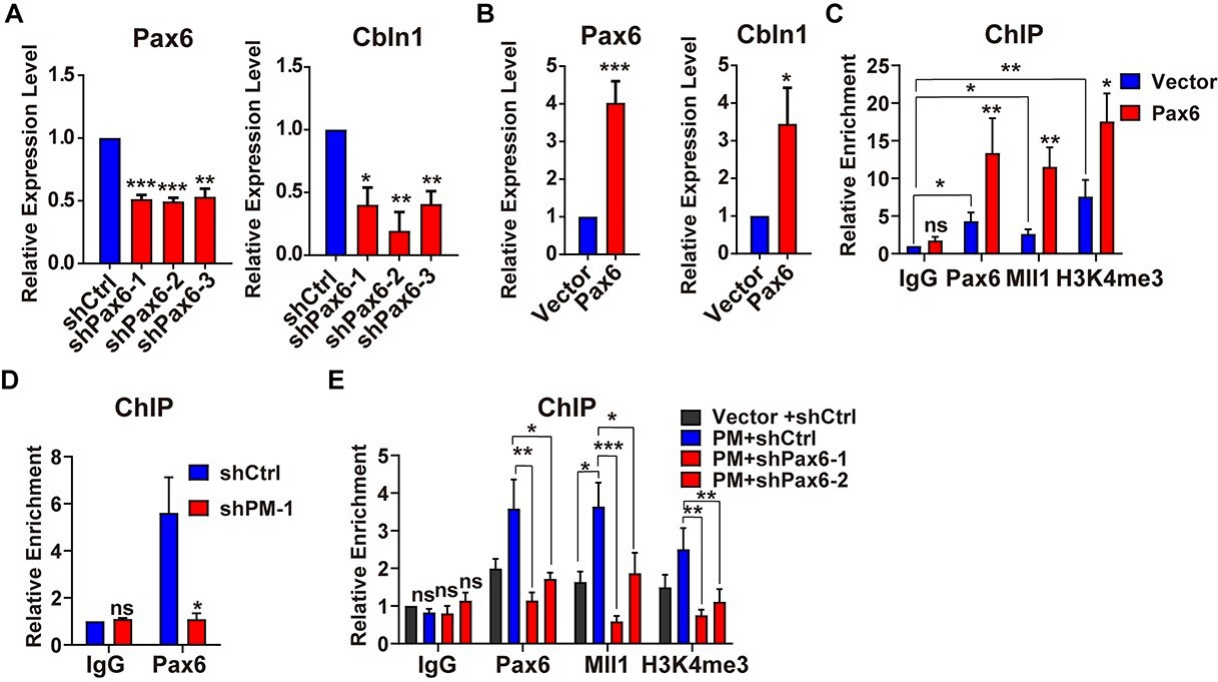
Pax6 is required for the *lncRNA-PM*-mediated Mll1-H3K4me3 recruitment. (A) The expression levels of *Pax6* and *Cbln1* mRNAs in the control or *Pax6* shRNAs-treated Neuro2a cells. All results were normalized to *Gapdh*. Data are shown as means ± SEMs, *n =* 3. (B) The expression levels of *Pax6* and *Cbln1* mRNAs in the control (Vector) or *Pax6*-overexpressed Neuro2a cells. All results were normalized to *Gapdh*. Data are shown as means ± SEMs, *n* = 3. (C) ChIP-qPCR detection of the indicated Pax6, Mll1, and H3K4me3 recruitment on the 5′ regulatory region of *Cbln1* in the control or *Pax6*-overexpressed Neuro2a cells. Data are shown as means ± SEMs, *n* = 3. (D) ChIP-qPCR detection of Pax6 on the 5′ regulatory region of *Cbln1* in the control or the indicated sh*PM* in Neuro2a cells. Data are shown as means ± SEMs, *n* = 3. (E) Changes of the Pax6, Mll1, and H3K4me3 deposition on the 5′ regulatory region of *Cbln1* in *PM*-overexpressed Neuro2a cells, with the treatments of Pax6 (shPax6-1 and shPax6-2) or control shRNAs (shCtrl). All the data of this figure can be found in the S1 Data file. Data are shown as means ± SEMs, *n* = 3. *P < 0.05, **P < 0.01, and ***P < 0.001. *Cbln1*, *Cerebellin-1*; ChIP-qPCR, chromatin immunoprecipitation couple with quantitative PCR; IgG, immunoglobulin G; *lncRNA-PM*, *lncRNA-Promoting Methylation*; ns, no significance.

We first confirmed the positive regulation of *Pax6* on *Cbln1* mRNA by either knockdown or overexpression of *Pax6* in Neuro2a cell (Fig 5A and 5B). ChIP assay showed that the recruitments of Mll1 and H3K4me3 were significantly increased in *Pax6*-overexpressed cells (Fig 5C). In order to rule out the possibility that *Pax6* regulated the expression level of *Mll1*, we performed RT-qPCR assay and found that the expression level of *Mll1* was not altered in *Pax6*-overexpressed cells (S6 Fig). Vice versa, *Mll1* siRNAs had no effects on the expression level of *Pax6* (S6 Fig). These results suggested that Pax6 activates *Cbln1* through Mll1-H3K4me3 axis.

To answer the question whether Pax6 was involved in *lncRNA-PM*-mediated Mll1-H3K4me3 activation of *Cbln1*, we checked whether the deposition of Pax6 was affected by modulation of *lncRNA-PM*. Our results showed that knocking down of *lncRNA-PM* significantly decreased the occupancies of Pax6 (Fig 5D), and overexpression of *lncRNA-PM* increased the occupancies of Pax6 (Fig 5E). In addition, knockdown of *Pax6* in the *lncRNA-PM*-overexpressed Neuro2a cells completely blocked the *lncRNA-PM*-induced recruitment of Mll1 and H3K4me3 on *Cbln1* (Fig 5E). Next, we conducted fractionationing experiments upon overexpression of *lncRNA-PM* in the Neuro2a cells and tested the changes of Pax6 and Mll1. Our results showed that *lncRNA-PM* promoted the DNA-bound fractions of both Pax6 and Mll1 (S7 Fig). Together with the results showed in Fig 4, our data suggested that *lncRNA-PM* activates *Cbln1* through recruitment of Pax6, Mll1, and H3K4me3. A possible underlying mechanism for such regulation is that *lncRNA-PM* helps to retain or translocate the chromatin associated fraction of Pax6 and Mll1.

### In vivo association of *lncRNA-PM* and Pax6/Mll1 complex with *Cbln1*

**Fig 6:**
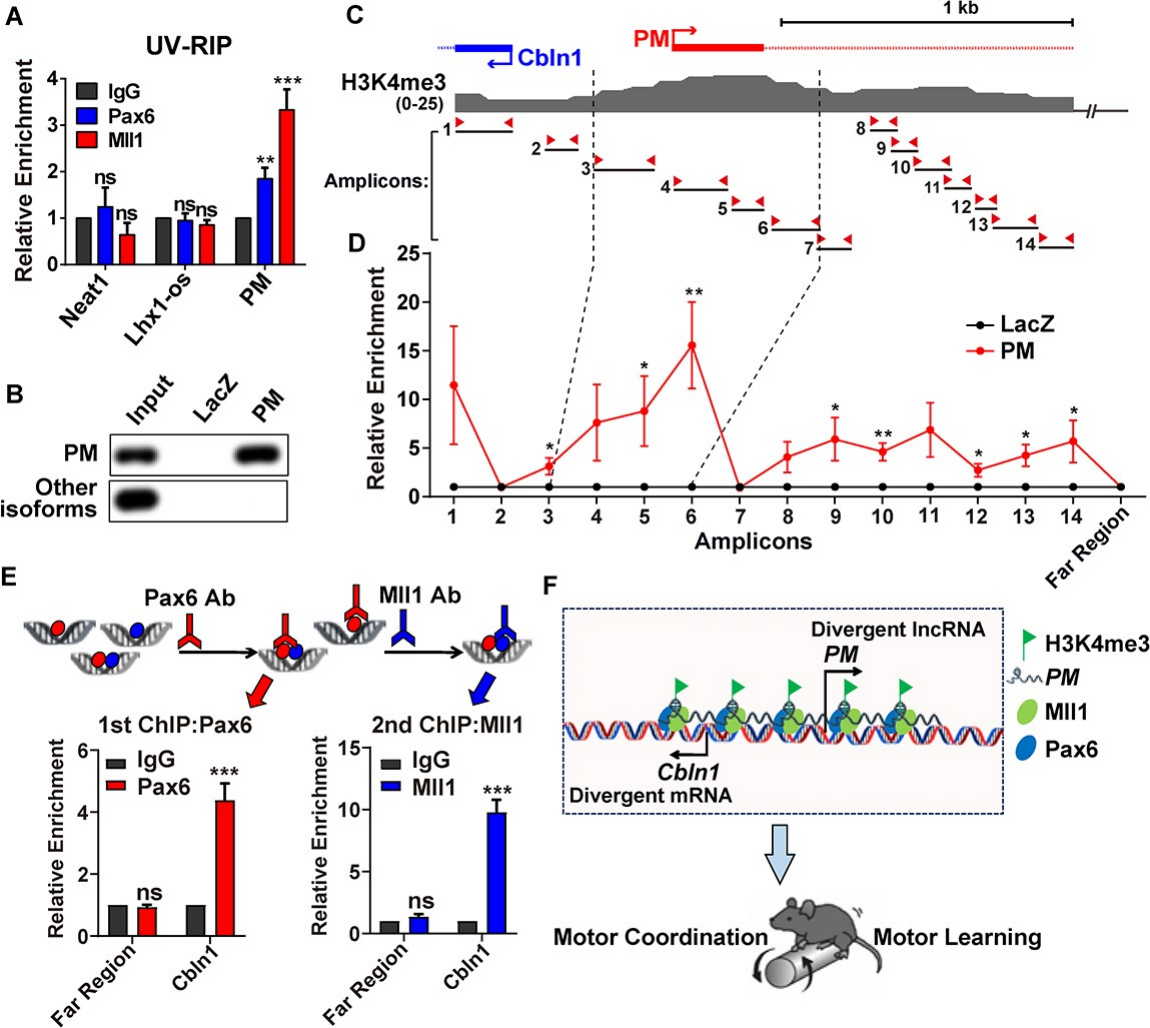
In vivo association of *lncRNA-PM* and Pax6/Mll1 complex with *Cbln1*. (A) UV-RIP assay showed *PM*:Pax6 and *PM*:Mll1 interactions in mouse cerebellum. *Neat1* and *lncRNA-lhx1os*: negative controls. Data are shown as means ± SEMs, *n =* 3. (B) RT-qPCR detection following *PM* RNA pull-down using cerebellum tissue. PM: *PM* probes; LacZ: control probes. (C) Illustration of H3K4me3 signature on the upstream regulatory region of *Cbln1* locus from +300 to −2,000 base pair based on ENCODE data in mouse cerebellum. The 14 pairs of amplicons are indicated. (D) ChIRP-qPCR showed occupancy of *PM* to the regions amplified by the indicated 14 amplicons and a control region that is 85 kb upstream of *Cbln1* transcription start site (Far Region). Data are shown as means ± SEMs, *n* = 3. (E) Re-ChIP of Pax6 and Mll1. Top: illustrated flowchart of double ChIP experiment. Bottom left: recruitments of Pax6 or IgG to *Cbln1* or the negative control region (Far Region) after first ChIP in mouse cerebellum. Bottom right: recruitments of Mll1 or IgG to *Cbln1* or Far Region after second ChIP in mouse cerebellum. Data are shown as means ± SEMs, *n* = 3. (F) Model: *LncRNA*-*PM* mediates the transcriptional activation of *Cbln1* through Pax6/Mll1-mediated H3K4me3 and maintains mouse motor coordination and motor learning. All the data of this figure can be found in the S1 and S2 Data files. *P < 0.05, **P < 0.01, and ***P < 0.001. *Cbln1*, *Cerebellin-1*; ChIP, chromatin immunoprecipitation; ChIRP, chromatin isolation by RNA purification; IgG, immunoglobulin G; *lncRNA-PM*, *lncRNA-Promoting Methylation*; ns, no significance; qPCR, quantitative PCR; Re-ChIP, Re-chromatin immunoprecipitation; RT-qPCR, reverse transcription-quantitative PCR; UV-RIP, UV-RNA immunoprecipitation.

In order to provide in vivo evidence of the above identified regulatory mechanism, we conducted a series of experiments in mouse cerebellum. Firstly, we applied the UV-RNA immunoprecipitation (UV-RIP) assay to detect binding of Mll1 and Pax6 to *lncRNA-PM*. We found that Mll1 and Pax6 were specifically associated with *lncRNA-PM* transcripts in the mouse cerebellum (Fig 6A). Serving as negative controls, unrelated *lncRNA-Neat1* and another cerebellum expressed *lncRNA-lhx1os* showed no interaction with either Mll1 or Pax6 (Fig 6A). Secondly, to answer whether *lncRNA-PM* is bound to the regulatory regions of *Cbln1* in cerebellum, we conducted chromatin isolation by RNA purification (ChIRP) experiments. To exclude the potential interference from other isoforms, we designed specific antisense oligonucleotides (ChIRP probes) of *lncRNA-PM* that did not overlap with other isoforms of *Gm2694*. These probes showed to be highly effective and specific in the mouse cerebellum extracts (Fig 6B). When compared to the negative control probes of lacZ, our ChIRP-qPCR analysis of *lncRNA-PM* revealed significant signals of *lncRNA-PM* to those upstream regulatory regions of *Cbln1* that are highly marked by H3K4me3 revealed by ChIP-seq (Fig 6C and 6D). Our ChIRP results suggested that *lncRNA-PM* is accumulating at its derived genomic locus to direct local histone modification changes. Consistently with these ChIRP results, our ChIP assays also revealed the *lncRNA-PM*-dependent recruitments of Pax6, Mll1, and H3K4me3 at multiple regions on the upstream regulatory regions of *Cbln1* (Figs 4 and 5, S8 Fig). Thirdly, in order to test whether Mll1 and Pax6 function as a complex on *Cbln1* in mouse cerebellum, we conducted Re-chromatin immunoprecipitation (Re-ChIP) assay and showed that Pax6 and Mll1 were simultaneously bound to *Cbln1* (Fig 6E). Taken all together, our data provided strong evidence of an association of *lncRNA-PM* and Pax6/Mll1 complex with the regulatory regions of *Cbln1* in cerebellum (Fig 6F).

### *LncRNA-PM* regulates genes in neuronal associated pathways

**Fig 7:**
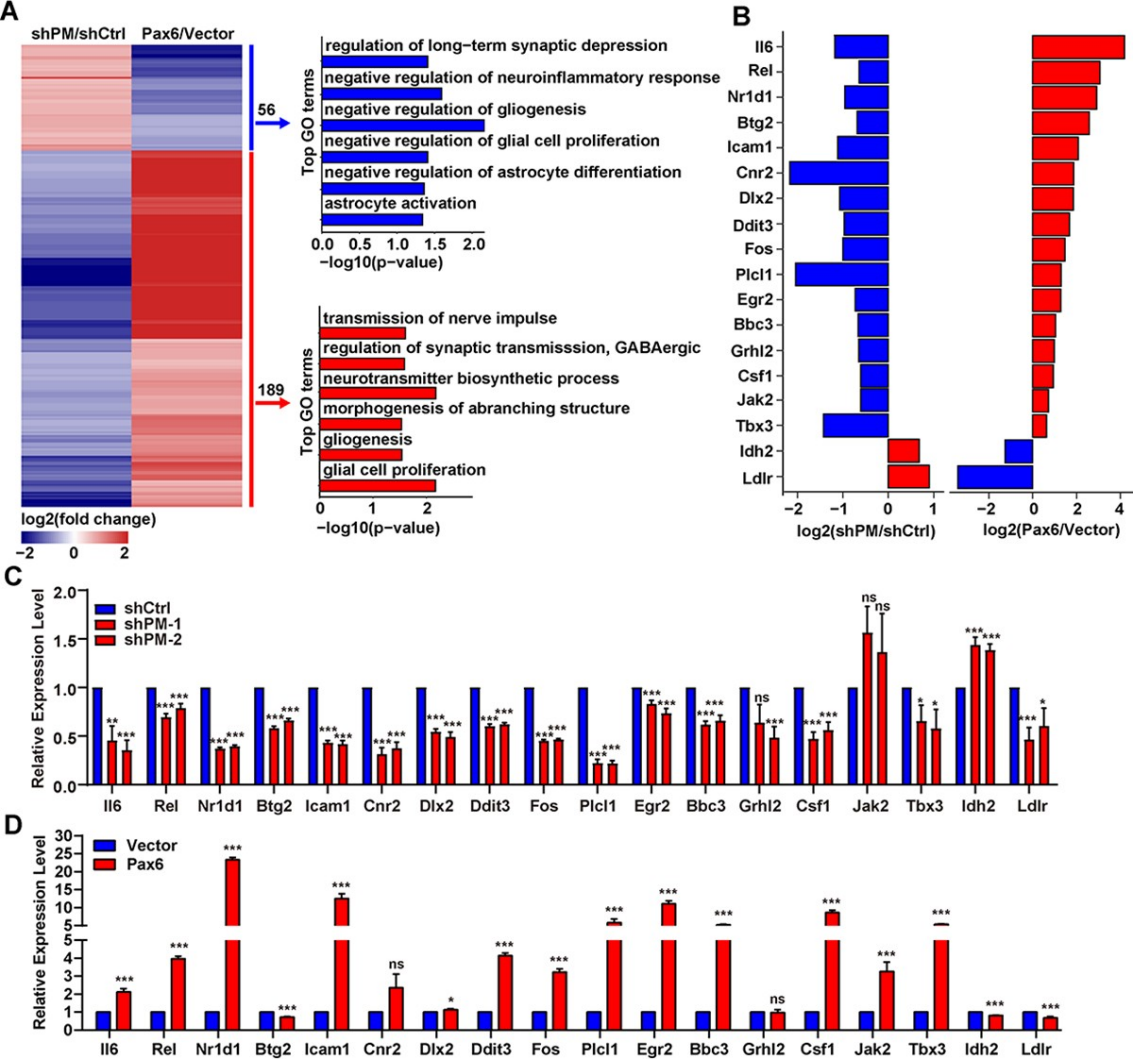
*LncRNA-PM* regulates neuronal associated genes. (A) Left: heatmap of dysregulated genes (|Fold change|>1.5, FDR < 0.05) in *PM* knockdown and *Pax6* overexpression RNA-seq. Right: GO biological processes of *PM* and *Pax6* coregulated genes. Red: positively regulated genes shared by *PM* and *Pax6*. Blue: negatively regulated genes shared by *PM* and *Pax6*. (B) Modulations of *lncRNA-PM* and *Pax6* impact neuronal associated genes. Expression changes for selected genes are shown as the log2 expression ratio. Red: positively regulated genes shared by *PM* and *Pax6*. Blue: negatively regulated genes shared by *PM* and *Pax6*. (C and D) qPCR detection of the indicated genes upon *PM* knockdown (C) and *Pax6* overexpression (D). All the data of this figure can be found in the S1 Data file. Data are shown as means ± SEMs, *n =* 3. *P < 0.05, **P < 0.01, and ***P < 0.001. FDR, false discovery rate; GO, Gene Ontology; *lncRNA-PM*, *lncRNA-Promoting Methylation*; ns, no significance; qPCR, quantitative PCR; RNA-seq, RNA sequencing.

Through analysis of the single-cell transcriptional study of the murine cerebellum, we found that *lncRNA-PM* exhibited a broader distribution than the *Cbln1* (S1 Fig). In addition, the shPM-injected mice resulted in defects on PC dendrites that were not observed in the *Cbln1*-null mice [7] (Fig 3C and 3D). These results suggested that *lncRNA-PM* acts as a broader cerebellar regulator. In order to gain a full scope of the genome-wide expression control of *lncRNA-PM*, we performed RNA-seq upon knockdown of *lncRNA-PM* by shPM-1 and shPM-2 and analyzed the common downstream targets from both shRNA transfectants. *LncRNA-PM* knockdown resulted in statistically significant changes in the expression levels of 504 genes (|fold change|>1.5) compared to the vector control; 352 (70%) of these genes were down-regulated and 152 (30%) were up-regulated. Importantly, we found that 245 (46.8%) of *lncRNA-PM*-regulated genes were also targeted by Pax6, identified from Pax6 overexpression RNA-seq (S9 Fig; S3 Table). The Gene Ontology (GO) analysis showed that these comprised 245 genes were significantly enriched in neuronal associated pathways, including GABAergic synaptic transmission, morphogenesis of branching structure, neurotransmitter biosynthetic process, and regulation of transmission of nerve impulse (Fig 7A). We further validated 14 downstream genes that were commonly regulated by *lncRNA-PM* and Pax6 in the same direction (Fig 7B–7D).

## Discussion

Cbln1 plays essential roles in developing and mature cerebellum [7,11]. Despite the exceptional high expression level of *Cbln1* in cerebellum, the molecular mechanism of *Cbln1* gene expression control is still unexplored. Recently, Krishnan V. and colleagues found that a ubiquitin-ligase and transcriptional coregulator *Ube3a* down-regulated *Cbln1* in several mouse brain regions and proposed a possible regulation at the transcriptional level of *Cbln1* [27]. In the present work, we found that *Cbln1* and its neighboring lncRNA *lncRNA-PM* are coexpressed in the cerebellar GCs. Our finding that *lncRNA-PM* activates the gene expression of *Cbln1* unveils the mystery of gene activation control of this critical cerebellar synapse organizer.

Pax6 has been recently identified as a cerebellar lineage marker [22,28]. In cerebellum, *Pax6* is highly expressed in GC layer of developing cerebellum and maintains in adult cerebellar GCs [29]. Loss of *Pax6* causes aberrant organization of GC layer, GC survival, and neurite extension [29]. Neural behavior analysis for *Pax6*-null mice/rats and investigation of clinical patients with Gillespie or WAGR syndrome indicated that *Pax6* is associated with cerebellar ataxia and other neurologic diseases [30–33]. Pax6 is known to interact with TrxG/MLL complex responsible for H3K4me3 [24]. In the present work, we demonstrated that the *lncRNA-PM*-mediated *Cbln1* activation is achieved through recruitments of Pax6, Mll1, and H3K4me3. Our findings provide a novel mechanism to explain the roles of Pax6 and Mll1 (via activating *Cbln1*) in cerebellum.

*LncRNA-PM*-mediated *Cbln1* activation occurs in both cultured cell system and the murine cerebellum. The *lncRNA-PM* knockdown mice exhibit similar phenotypes as the *Cbln1*-null mice in synaptic integrity and motor function. Intriguingly, the *Cbln1*-null mice expressed a higher density of VGluT2 and showed no overt difference in PC dendrites, while significant reductions of VGluT2 and Calbindin signals have been found in our *lncRNA-PM* knockdown mice. Such discrepancies may be resulted from 2 aspects: the different methodology and animal stages utilized for generating the 2 types of animal models and the broader distribution of *Gm2694* than *Cbln1* in the cerebellum. First, the reported *Cbln1*-null mice were generated by replacing the *Cbln1* gene with *Cbln1* promoter-driven LacZ. Thus, any impacts resulted from *Cbln1* depletion would have been present throughout the course of development. However, some impacts also might be compromised during development. Distinct from those *Cbln1*-null mice, our study used 2-month-old adult mice for the *lncRNA-PM* knockdown. It is well known that synapse remodeling of diverse fiber types to their destination neurons is highly dynamic in the developing and mature cerebellum. For example, during the first 3 postnatal weeks, redundant climbing fibers (marked by VGluT2) are eliminated, culminating in a situation where the majority of PCs are innervated by a single climbing fiber [34,35]. Second, *Gm2694* is evident in GABAergic progenitors, interneurons, and glutamatergic cerebellar nuclear neurons, in addition to the coexpression with *Cbln1* in the cerebellar GCs.

Increasing the number of isoforms is a general strategy used by different types of cells to expand the transcriptome diversity. For instance, in the CNS, alternative splicing-generated mRNA isoforms are important in the development, migration, synaptic transmission, and plasticity of neurons [36,37]. LncRNAs have also been recently reported as targets of alternative splicing [36–42]. Although a few studies have shown that lncRNAs participate in the dynamic regulation process of alternative splicing in CNS [43,44], it is currently largely unclear about the biological significance of lncRNA isoforms in CNS. Our identification that *Gm2694* regulates *Cbln1* transcriptional activation through an isoform-specific manner expands the current understanding on the specificity mediated by lncRNA isoforms.

Collectively, our work provides a previously unidentified mechanism of *lncRNA-PM*-Pax6-Mll1-H3K4me3-mediated *Cbln1* activation and expands our understandings on the biological roles of lncRNA in the cerebellum. Our observation that *lncRNA-PM* functions in a different way than other *Gm2694* isoforms sheds light on the biological significance of lncRNA-based isoform in nervous system.

## Materials and methods

### Animal ethics statements

All animal experiments were approved and carried out in accordance with the Institutional Animal Care and Use Committee of the University of Science and Technology of China (permission number: USTCACUC1801023) and the National Institutes of Health Guide for the Care and Use of Laboratory Animals.

### Cell culture and transfection

The HEK 293T (ATCC, USA) and Neuro2a cells (ATCC, USA) were maintained in Dulbecco’s modified Eagle’s medium (DMEM) (Gibco, USA) containing 10% fetal bovine serum (FBS) (Gibco, USA), 1% penicillin–streptomycin (WISENT, Canada), and 1% L-glutamine. Transfections were conducted using Lipofectamine 3000 (Invitrogen, USA) according to the manufacturer’s instructions.

### SiRNAs and shRNAs

Three siRNAs of *Mll1* were designed and synthesized from Ribobio (Guangzhou, China). Three independent shRNAs specifically targeting 3 different regions of *lncRNA-PM* or *Pax6* were designed and separately cloned into pLKO.1 vector to generate 3 *lncRNA-PM* or *Pax6* shRNAs. Two of 3 *lncRNA-PM* shRNAs inserted pLKO.1 vector were separately cloned into pAAV-CAG-eGFP-U6-shRNA vector (OBIO, China) to generate 2 pAAV-CAG-eGFP-U6-sh*PM* vectors. The primers are listed in S4 Table.

### Plasmids construction

The ectopic expression constructs of 5 *Gm2694* isoforms (*ENSMUST00000180700*.*8*/*Gm2694-201*, *ENSMUST00000181898*.*1*/*Gm2694-204*/*lncRNA-PM*, *ENSMUST00000182174*.*7*/*Gm2694-205*, *ENSMUST00000182758*.*2/Gm2694-207*, and *ENSMUST00000211631*.*1*/*Gm2694-211*) were amplified from mouse cerebellum cDNA and cloned into the pZW1-sno vector [45], respectively. The *Pax6* (Gene ID: 18508) expression plasmid was constructed by cloning Pax6 ORF from mouse cerebellum cDNA and inserting it into the multiple cloning site (MCS) of the p3xflag-Myc-CMV-24 vector (Sigma, USA).

### RNA extraction and reverse transcription-quantitative PCR (RT-qPCR)

Total RNA of cultured cell or mouse brain tissues was isolated using the TRizol Reagent (Ambion, USA), treated with RNase-free DNase I (Thermo Scientific, USA). Reverse transcription was performed using HiScript II One Step RT-PCR Kit (Vazyme, China) according to the manufacturer’s instructions. qPCR was performed using SYBR Green reagents (Vazyme, China) in Light Cycle 96 (Roche, USA). Relative gene expression was calculated using the 2^−ΔΔCt^ method, and *Gapdh* served as an internal control. The primers are listed in S5 Table.

### Rapid amplification of cDNA ends (RACE)

RACE was conducted using the 5′ RACE System and 3′ RACE System Kit (Invitrogen, USA) following the manufacturer’s instructions. Briefly, for the 5′ RACE, cerebellum RNA was reverse transcribed with the antisense gene specific primers (GSPs) to *lncRNA-PM* or *Cbln1*, and the first-strand cDNA product was homopolymeric tailed. Two rounds of nest PCR amplification were then performed with sense generic primer and antisense GSPs (Flexcycler, Germany). For the 3′ RACE, the cerebellum RNA was reverse transcribed using the adapter primer containing a 3′ stretch of poly(dT). Amplification was sequentially performed by nest PCRs using oligonucleotides to the non-poly(dT) portion of the adapter primer and the GSPs specific to *lncRNA-PM* or *Cbln1*. The PCR products were subjected to electrophoresis, purification, and DNA sequence analysis. The GSPs used in RACE experiments are as follows:

5′ RACE:

Reverse transcription:

Cbln1: 5′ - AAACTGTAGATGCCTT-3′;

lncRNA-PM: 5′ - CAAGTGAAGACCATAC-3′;

First-round PCR amplification:

Cbln1: 5′ - GTGCTGCGTTCTGAGTCAAA-3′;

lncRNA-PM: 5′ - TCCTTCCCTGGTCCCTTTAC-3′;

Second round of amplification:

Cbln1: 5′ - TCCAGAACAAATGTCCCCGC-3′;

lncRNA-PM: 5′ - CAGGCCATGAGAACACTGTG-3′;

3′ RACE:

First-round PCR amplification:

Cbln1: 5′ - GGTGACTGGTTTGACTATAC-3′;

lncRNA-PM: 5′ -TGAAACTACATCCACGCCCA-3′;

Second round of amplification:

Cbln1: 5′ - AGGCTGCTTGGTGTTTGTTC-3′;

lncRNA-PM: 5′ - GCGTATGGTCTTCACTTGCC-3′.

### Dual luciferase reporter assay

The 5′ regulatory sequences of *Gm2694* and *Cbln1* were amplified using PCR from mouse cerebellum genomic DNA and sequentially ligated into pGL3-basic vector to generate pGL3-*Gm2694* or pGL3-*Cbln1* reporter plasmid. To measure the reporters’ activity, either pGL3-basic vector, pGL3-*Gm2694*, or pGL3-*Cbln1* plasmid was cotransfected into HEK 293T cells together with Renilla luciferase reporter plasmid. To measure *lncRNA-PM* effect on the pGL3-*Cbln1*, either pZW1-sno vector or pZW1-sno-*lncRNA-PM* plasmid was cotransfected into HEK 293T cells together with pGL3-*Cbln1* and Renilla luciferase reporter plasmid. Two days after transfection, firefly and Renilla luciferase activity were measured by a dual luciferase reporter assay system (GeneCopoeia, USA). The data are represented as mean ± SEM of 3 independent experiments.

### Cerebellum *lncRNA-PM* knockdown mice

Mice were group housed in groups of 2 to 5 with a 12-h light/dark cycle (lights off at 6 PM) and free access to food and water ad libitum under specific pathogen-free conditions. Male C57BL/6J mice were purchased from Beijing SPF Biotechnology limited company and used for all experiments. For pAAV-CAG-eGFP-U6-shRNA (OBIO, China) injection, 3 AAV-based plasmids (AAV NC, AAV sh*PM*-1, or AAV sh*PM*-2) were respectively injected into normal 2-month mice to produce 3 groups of experimental mice. Briefly, glass micropipettes attached to 10 μl syringes were placed bilaterally into the cerebellum (A/P: −7.05 mm; M/L: ±1.2 mm; D/V: −1.6 mm), and 1 μl of virus was infused to each hemisphere at a rate of 50 nl/min using a pump (KD Scientific, USA). Mice were allowed to recover for 2 months before behavioral tests or immunofluorescence.

### Cylinder test

The cylinder test was designed to assess forepaw preference on mice and conducted in a transparent cylinder. A video recorder was placed behind the explorative area to allow recording forelimb movements in all directions. The animals were not habituated to the cylinder prior to filming. The forepaw use was counted and normal-impaired/all was used a quantitative outcome parameter. Seven mice were tested in each group.

### Rotarod test

We used the rotarod training system (Softmaze, China) to test the motor coordination and motor learning skill of mice. Briefly, before the first testing sessions, the mice were habituated to stay on a stationary rod for 2 min. To assess motor skill, a total of 9 trials for the rotarod test were carried out using an accelerating protocol from 0 to 60 rpm in 300 s with 30-min intertrial intervals. Three testing trials per day were performed for 3 consecutive days. After falling, the mice were immediately placed back to their home cages, and the time to fall was automatically recorded by the rotarod software. Six male mice in each group were used for the experiment. Six mice in each group were divided into 2 experiments, and 3 mice were simultaneously tested.

### Brain tissue preparation

Mice were deeply anesthetized by intraperitoneal (IP) injection of pentobarbital (40 mg/kg), and then perfused with chilled PBS, followed by 4% paraformaldehyde (PFA) (Biosharp, China). Brains were removed and immersed in 4% PFA overnight, and subsequently incubated in 30% (wt/vol) sucrose in PBS at 4°C for 18 h. Sagittal brain sections with 25 μm thickness were made using a cryostat (Leica CM1950, Germany) at −20°C and conducted for desired experiments.

### Immunofluorescence (IF) and FISH

Cultured Neuro2a cells growing on coverslips were fixed by 4% PFA for 10 min and subsequently incubated with 0.2% Triton X-100 in PBS. After 30 min of blocking using 1% BSA in PBST (0.05% Tween-20 in PBS), Neuro2a cells were incubated with rabbit anti-Cbln1 antibody (Novus, NBP1-17239, 1:200) in PBST overnight at 4°C. On the following day, the cells were washed with PBST and incubated with rhodamine-labeled secondary antibody (1:100) for 30 min at room temperature on a shaker. After PBST wash and stained with DAPI for 10 min, the coverslips were sealed with 75% glycerin and subjected to laser scanning confocal microscope (ZEISS710, Germany).

For brain sections, sections were incubated with 0.5% Triton X-100 in PBS for 15 min after wash (3 times of PBS, each time 5 min). After 40 min of blocking (3% albumin from bovine serum, 3% concentrated goat serum in PBS), the sections were incubated with rabbit anti-Cbln1 antibody (Abcam, ab64184, 1:50), rabbit anti-H3K27ac antibody (Abcam, ab4729, 1:100), mouse anti-Calbindin antibody (Abcam, ab82812, 1:100), rabbit anti-VGluT1 (Abcam, ab227805, 1:100), or rabbit anti-VGluT2 antibody (Abcam, ab216463, 1:100) in PBS at 4°C for 24 h. On the following day, the sections were washed in PBS and incubated with rhodamine- or Alexa Fluor 647–labeled secondary antibody (1:100) for 30 to 60 min.

FISH assays were performed using FISH Kits (Genepharma, China) according to the manufacturer’s instructions. *lncRNA-PM* and *Cbln1* probes were designed by Biosearch Technologies website (https://www.biosearchtech.com/stellarisdesigner/). All probes were synthesized from General Biosystems, China (Probes for *Cbln1*: 5′-TACGATGGGCTCTGTCTCAT-3′, 5′-TTCAACATGAGGCTCACCTG-3′, 5′-GAAGGTTGAGTACTTCCAGC-3′; Probes for *lncRNA-PM*: 5′-TTTTGTATGCATGCTGACCG-3′, 5′-CTCAGGGAAGGTCTTCTTAA-3′, 5′-GTTTATTATTCCTTGTGGAG-3′). Laser scanning confocal microscope (ZEISS710, Germany) was used for images.

To quantify the signal intensity obtained from the above described IF and FISH images, we followed a previously reported analytic procedure with some modifications [11]. Briefly, 3 square images of 40 × 40 μm were taken within the cerebellar molecular layer. All images were obtained by exposures and gains at fixed parameters for each fluorescent channel. The images were analyzed using ImageJ to evaluate the signal intensity. After subtracting the background signals, the values of signal intensity from 3 regions were averaged to represent the data for each treatment, and the experiment was performed 3 times independently. All the comparable treatments were simultaneously performed and analyzed on the same day.

### Electron microscopy

Mice were perfused with 2% PFA and 2.5% glutaraldehyde. Cerebellum were sliced sagittally and fixed with 2.5% glutaraldehyde. The cerebellum slices were postfixed with 1% osmium acid, dehydrated through graded acetone, and embedded in epoxy resin. Ultrathin sections (70 nm) were made from the epoxy resin blocks using an ultramicrotome (Leica UC7, Germany) and stained with uranium acetate and lead citrate. Micrographs were taken by electron microscope (FEI, USA) at 8,200×.

### Western blot

The cells were washed in PBS and lysed with RIPA buffer (Vazyme, China) supplemented with protease inhibitor cocktail (Roche, Germany). After quantification using One DropTM 1000+ (ANTPEDIA, China), proteins were separated by SDS-PAGE under denaturing conditions and transferred to PVDF membranes (Millipore, USA). Membranes were blocked with 5% fat-free milk for 2 h at room temperature and incubated with primary antibodies for Gapdh (Proteintech, 60004-1-Ig, 1:5,000), Mll1 (CST, D668N, 1:1,000), Mll2 (CST, E6A8V, 1:1,000), Pax6 (Abcam, ab5790, 1:500), and H2b (Abcam, ab52599, 1:10,000). The membranes were then incubated with secondary antibodies conjugated with horseradish peroxidase (Proteintech, USA). Protein signals were visualized using the ECL detection reagent (Thermo Scientific) on UVP (Analytik Jena AG, Germany).

### Subcellular fractionation assay

Neuro2a cells (7 × 10^6^) were lysed in RSB-100 buffer (100 mM Tris-HCl (pH 7.4); 2.5 mM MgCl2; 100 mM NaCl; 40 μg/mL digitonin) at 4°C for 15 min. After centrifugation at 2,000*g* for 8 min, the supernatant was collected as the cytosolic fraction and saved for western blot. The pellets were resuspended in RSB-100T buffer (RSB-100 containing 0.5% Triton X-100) and rotated at 4°C for 15 min. After centrifugation at 2,000*g* for 8 min, the supernatant was collected as the nuclear fraction and saved for western blot. The pellets were resuspended in RSB-100T buffer and subjected to sonication (Scientz, China). After centrifugation at 4,000*g* for 15 min, the soluble DNA-bound fraction was collected and subjected for western blot with the previously obtained cytosolic and nuclear fraction.

### UV-RNA immunoprecipitation (UV-RIP), chromatin immunoprecipitation (ChIP), and Re-ChIP

For the UV-RIP assay, dounced cerebellum tissues were irradiated with UV light (400 mJ/cm^2^). After centrifuged at 16,000 rpm at 4°C for 10 min, the cerebellum tissues were lysed for 30 min in RIPA buffer supplemented with RNase inhibitors (Beyotime, China) and protease inhibitor cocktail. After centrifuged at 16,000 rpm for 10 min at 4°C, the supernatant was precleared with 25 μl of Protein A/G Plus-Agarose beads (Santa Cruz Biotechnology, USA) at 4°C for 2 h. Then, the precleared lysate was incubated with 50 μl of Protein A/G Plus-Agarose beads that were precoated with 4 μg of IgG (Abcam, 172730), Pax6 (Abcam, ab5790), or Mll1 (Sigma, PLA0100) antibody. After 12-h incubation, the beads were washed 5 times using RIPA buffer. Finally, RNA was extracted using TRizol Reagent (Ambion, USA). The protein-bound RNA was analyzed by RT-qPCR.

The ChIP assay was performed as previously reported [46]. Briefly, dounced cerebellum tissues or cultured cells were cross-linked with 1% formaldehyde and neutralized with 125 mM glycine. After cell lysing, the lysates were sonicated. Supernatants were then incubated with 2 μg desired antibodies for IgG (Abcam, 172730), H3K4me1 (Abcam, ab8895), H3K4me3 (Abcam, ab8580), H3K27ac (Abcam, ab4729), H3K27me3 (Abcam, ab6002), Mll1 (Sigma, PLA0100), or Pax6 (Abcam, ab5790) at 4°C overnight. Immunoprecipitated complexes were collected using Protein A/G PLUS-Agarose beads (Santa Cruz Biotechnology, USA). The isolated chromatin fraction was purified by EasyPure Genomic DNA Kit (TransGene Biotech, Beijing, China). For Re-ChIP experiments, complex obtained from the first ChIP were eluted by incubation for 30 min at 37°C in 25 μl 10 mM DTT. After centrifugation, the supernatant was diluted 20 times with Re-ChIP buffer (1% TritonX-100, 2 mM EDTA, 20 mM Tris-HCL, 150 mM NaCl (pH 8.1)) and subjected again to the ChIP procedure. The DNA product was analyzed by qPCR. For the data analysis, the control IgG group of each set of data was used as the normalizer for the following antibody groups to determine the binding specificity of the desired antibody. The related primers are listed in S6 Table.

### Chromatin isolation by RNA purification (ChIRP)

Antisense RNA oligonucleotides (probes) for lncRNA-PM and LacZ (negative control) were designed by Biosearch Technologies website (https://www.biosearchtech.com/stellarisdesigner/) and synthesized with 3′-biotin-TEG modification (Sangon, China). Probes targeting to each tested RNA molecule were pooled and diluted to a stock concentration of 100 μM. The ChIRP assay was performed as previously reported with some modifications [47]. Briefly, 5 dounced mouse cerebellum were cross-linked with 3% formaldehyde for 30 min at room temperature on a shaker, and the reaction was quenched with 0.125 mM glycine for 5 min. Cells were collected by spinning at 2,000*g* for 5 min, and nuclei were collected with Covaris truChIP Chromatin shearing tissue kit (Covaris, USA) supplemented with RNase inhibitors (Beyotime, China) and protease inhibitors (Roche, USA). Nuclei were immediately sonicated by Covaris M220 (Covaris, USA) to 200 to 500 bp fragments and verified by gel electrophoresis. The sonicated samples were spun at 16,100*g* for 10 min at 4°C, and the supernatant was then transferred to a new tube. Before continuing to the next steps, 2 sets of 10% supernatant were removed into 2 separate tubes and saved as DNA or RNA input control, respectively. Double volume of hybridization buffer and the desired probes (1 μl of 100 μM probes per 1 ml of lysate) were added to the remaining supernatant and incubated at 37°C for 6 h in a rotator. After incubation, prewashed Dynabeads MyOne Streptavidin C1 magnetic beads (Invitrogen, USA) (100 μl of beads per 1ml of lysate) were added and incubated for 30 min at 37°C on a rotator. The Dynabeads were then captured by magnets (Invitrogen, USA) and washed with Wash Buffer for 5 min at 37°C for a total of 5 times. After the last wash, beads were resuspended in 1 ml Wash Buffer. A volume of 100 μl was taken for RNA isolation, and the rest was used for DNA isolation. RNA enrichment was confirmed by RT-qPCR. RNA-bound DNA was analyzed by qPCR. For the data analysis, the LacZ control was used as the normalizer to determine the binding specificity to each tested region. The related primers and probes are listed in S6 and S7 Tables.

### RNA-seq

RNA library construction and sequencing were performed with BGISEQ-500 platform. For each experiment, 3 biological repeats were sequenced. The differential expressed genes (DEGs) were determined by using |fold change| >1.5.

### Quantification and statistical analysis

Statistical analysis was carried out using Microsoft Excel software and GraphPad Prism to assess the differences between experimental groups. Statistical significance was analyzed by two-tailed Student *t* test and expressed as a *P* value. Especially, two-way ANOVA test was performed to measure significance of interaction in rotarod test. *P* < 0.05 were considered to be statistical significance.

The numerical data used in all figures are included in S1 Data. The raw images of blot and gel used in all figures are included in S2 Data.

## Supporting information

S1 Fig Distributions of *Gm2694*, *Pax6*, and *Cbln1* revealed by cerebellum single-cell RNA-seq datasets. (A) t-SNE plot displaying the distribution of the main cerebellar cell types. (B) The t-SNE distributions of *Gm2694*, Pax6, and *Cbln1*. The data used for these analyses are from [10]. *Cbln1*, *Cerebellin-1*; RNA-seq, RNA sequencing; t-SNE, t-Stochastic Neighbor Embedding.
(TIF)

S2 Fig RACE and sequence of *lncRNA-PM* and *Cbln1*. (A) Electrophoresis gel images of the 5′ and 3′ RACE results of *Cbln1*. (B) Sequence of *Cbln1*. (C) Electrophoresis gel images of the 5′ and 3′ RACE results of *lncRNA-PM*. (D) Sequence of *lncRNA-PM*. All the data of this figure can be found in the S2 Data file. *Cbln1*, *Cerebellin-1*; *lncRNA-PM*, *lncRNA-Promoting Methylation*; RACE, rapid amplification of cDNA ends.
(TIF)

S3 Fig The relative expression levels of the 12 nearby genes of the *Cbln1/Gm2694* locus upon *lncRNA-PM* knockdown in Neuro2a cells. (A) Illustration of the *Cbln1*, *Gm2694*, and the tested 12 nearby genes. (B) Relative expression levels of the indicated 12 genes upon the indicated treatments in Neuro2a cells. All the data of this figure can be found in the S1 Data file. Data are shown as means ± SEMs, *n =* 3. *P < 0.05, **P < 0.01, and ***P < 0.001. *Cbln1*, *Cerebellin-1*; *lncRNA-PM*, *lncRNA-Promoting Methylation*.
(TIF)

S4 Fig Image of eGFP showed wide distribution of virus infection in the mouse cerebellum. eGFP, enhanced green fluorescent protein.
(TIF)

S5 Fig *LncRNA-PM* increased the recruitment of Mll1, but not Mll2-Mll4, to *Cbln1*. ChIP-qPCR detection of Mll1, Mll2, Mll3, and Mll4 on the indicated upstream regulatory regions of *Cbln1* in the control or *PM*-overexpressed Neuro2a cells. All the data of this figure can be found in the S1 Data file. Data are shown as means ± SEMs, *n* = 3. ***P < 0.001. *Cbln1*, *Cerebellin-1*; ChIP, chromatin immunoprecipitation; IgG, immunoglobulin G; *lncRNA-PM*, *lncRNA-Promoting Methylation*; ns, no significance; qPCR, quantitative PCR.
(TIF)

S6 Fig **The relative expression levels of *Mll1* (A) and *Pax6* (B) under the indicated treatments in Neuro2a cells.** All results were normalized to *Gapdh*. All the data of this figure can be found in the S1 Data file. Data are shown as means ± SEMs, *n* = 3. ns, no significance.
(TIF)

S7 Fig *LncRNA-PM* promoted the DNA-bound fractions of Pax6 and Mll1. (A) Representative images of the effect of *lncRNA-PM* on the protein levels of Pax6 and Mll1 in the whole cell lysates (Input), cytoplasm (Cyto), nucleoplasm (Nucl), and DNA-bound fractions. (B) Quantification of A. Gapdh and H2b are markers for cytoplasm and nucleus fractions, respectively. All the data of this figure can be found in the S1 and S2 Data files. Data are shown as means ± SEMs, *n* = 3. *P < 0.05 and **P < 0.01. *lncRNA-PM*, *lncRNA-Promoting Methylation*; ns, no significance.
(TIF)

S8 Fig *LncRNA-PM*-dependent recruitments of Pax6, Mll1, and H3K4me3 to the upstream regulatory region of *Cbln1* (amplicon 3). (A, B) Occupancies of H3K4me3 and H3K27ac (A) and Mll1 (B) on the 5′ regulatory region of *Cbln1* detected by amplicon 3 (indicated in Fig 6C) in the control (Vector) or *PM*-overexpressed (PM) Neuro2a cells. Data are shown as means ± SEMs, *n* = 3. (C) Occupancies of Mll1 and Pax6 on the 5′ regulatory region of *Cbln1* detected by amplicon 3 (indicated in Fig 6C) in the control or the indicated shPMs in Neuro2a cells. Data are shown as means ± SEMs, *n* = 3. (D) Occupancies of the Mll1 and H3K4me3 on the 5′ regulatory region of *Cbln1* detected by amplicon 3 (indicated in Fig 6C) in *PM*-overexpressed Neuro2a cells, with the treatments of Mll1 (siMll1-1 and siMll1-2) or control siRNAs (siCtrl). All the data of this figure can be found in the S1 Data file. Data are shown as means ± SEMs, *n* = 3. *P < 0.05, **P < 0.01, and ***P < 0.001. *Cbln1*, *Cerebellin-1*; ChIP, chromatin immunoprecipitation; IgG, immunoglobulin G; *lncRNA-PM*, *lncRNA-Promoting Methylation*; ns, no significance.
(TIF)

S9 Fig Venn diagram of the *lncRNA-PM* (*PM*) and *Pax6* coregulated genes. (A) Venn diagram of the positively regulated genes shared by *PM* and *Pax6*. (B) Venn diagram of the negatively regulated genes shared by *PM* and *Pax6*. *lncRNA-PM*, *lncRNA-Promoting Methylation*.
(TIF)

S1 Table List of 57 lncRNAs that are abundantly expressed in the cerebellum. (DOCX)

S2 Table List of ChIP-seq data files. (DOCX)

S3 Table List of 245 genes coregulated by *lncRNA-PM* and Pax6 in RNA-seq. (XLSX)

S4 Table List of shRNAs and siRNAs. (DOCX)

S5 Table List of RT-qPCR primers. (DOCX)

S6 Table List of qPCR primers for ChIP and ChIRP. (DOCX)

S7 Table List of ChIRP probes. (DOCX)

S1 Data Excel spreadsheet containing, in separate sheets, the underlying numerical data, and statistical analysis for figure panels 1A, 1B, 1C, 1D, 1E, 1F, 1G, 1H, 1J, 2E, 2F, 2G, 2H, 2I, 2K, 2L, 3D, 4B, 4C, 4D, 4E, 4F, 4G, 5A, 5B, 5C, 5D, 5E, 6A, 6D, 6E, 7A, 7B, 7C, 7D, S3B, S5, S6A, S6B, S7B, S8A, S8B, S8C, and S8D. (XLSX)

S2 Data Raw images of blot and gel for figure panels 6B, S2A, S2B, and S7A. (PDF)

## Abbreviations

*Cbln1*: *Cerebellin-1*
ChIP: chromatin immunoprecipitation
ChIRP: chromatin isolation by RNA purification
CNS: central nervous system
DEG: differential expressed gene
DMEM: Dulbecco’s modified Eagle’s medium
FBS: fetal bovine serum
FISH: fluorescence in situ hybridization
GC: granule cell
GluD2: glutamate receptor delta2
GO: Gene Ontology
GSP: gene specific primer
IP: intraperitoneal
lncRNA: long noncoding RNA
*lncRNA-PM*: *lncRNA-Promoting Methylation*
MCS: multiple cloning site
Nrx: neurexin
PC: Purkinje cell
PF: parallel fiber
PFA: paraformaldehyde
PSD: postsynaptic density
RACE: rapid amplification of cDNA ends
Re-ChIP: Re-chromatin immunoprecipitation
RNA-seq: RNA sequencing
RT-qPCR: reverse transcription-quantitative PCR
UV-RIP: UV-RNA immunoprecipitation
VGluT1: vesicular glutamate transporter 1
