# Supplementary figures and images for: Long noncoding RNA PM maintains cerebellar synaptic integrity and Cbln1 activation via Pax6/Mll1-mediated H3K4me3
Source: PLoS Biol. 2021 Jun 10;19(6):e3001297. doi: 10.1371/journal.pbio.3001297 (PMC8219131; doi:10.1371/journal.pbio.3001297)

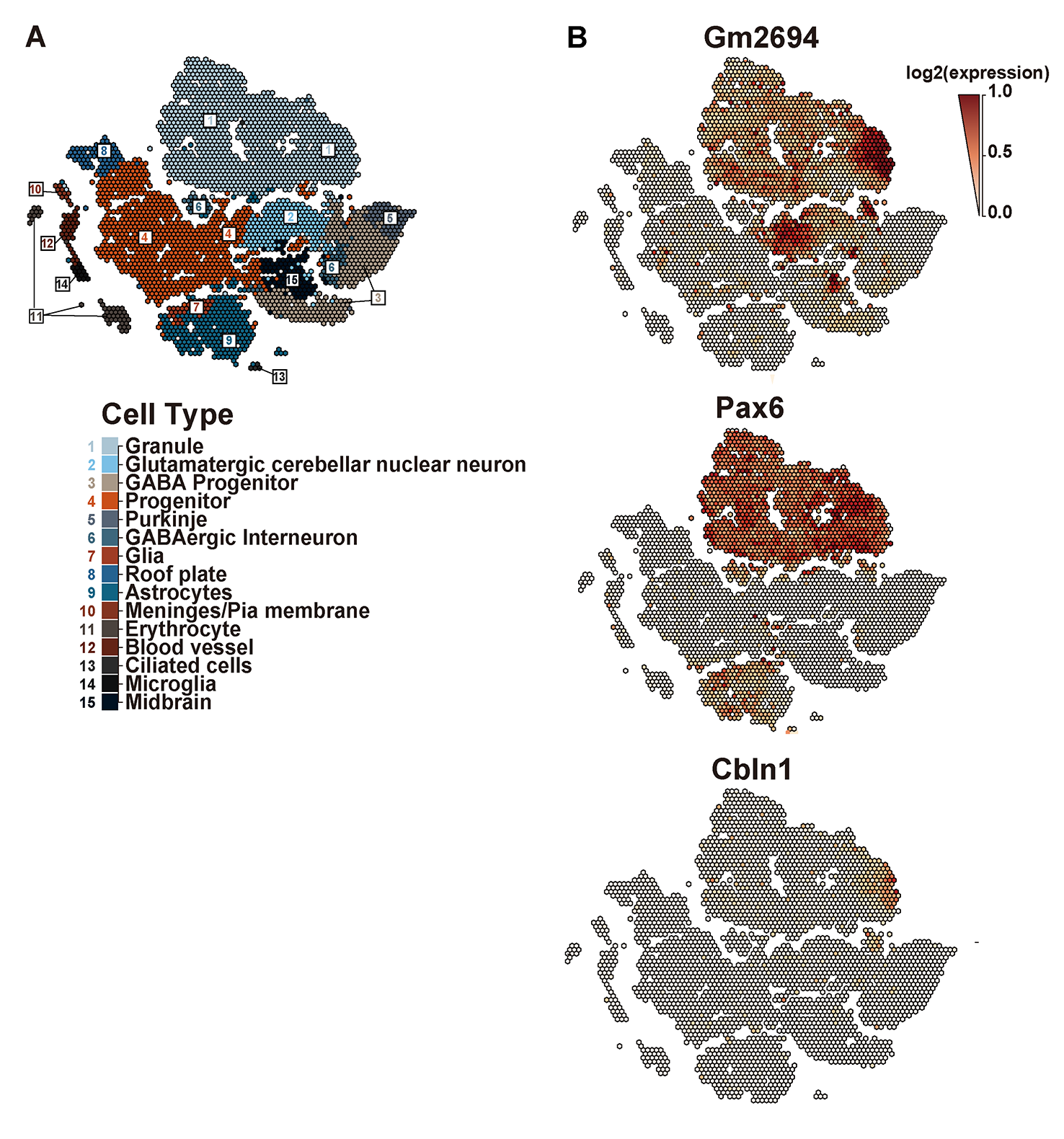

Supplement: S1 Fig — (A) t-SNE plot displaying the distribution of the main cerebellar cell types. (B) The t-SNE distributions of Gm2694, Pax6, and Cbln1. The data used for these analyses are from [10]. Cbln1, Cerebellin-1; RNA-seq, RNA sequencing; t-SNE, t-Stochastic Neighbor Embedding. (TIF) [file pbio.3001297.s001.tif]

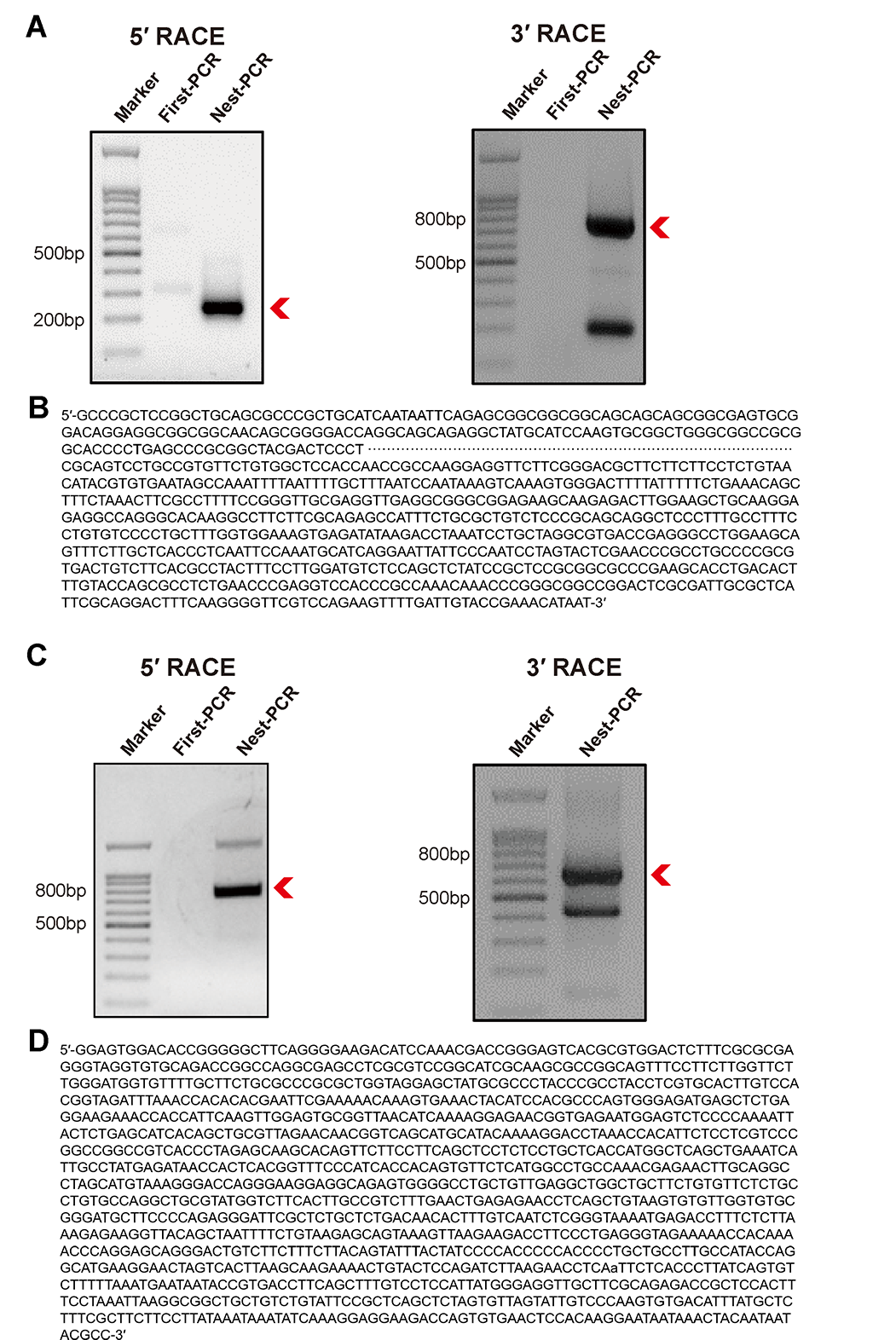

Supplement: S2 Fig — (A) Electrophoresis gel images of the 5′ and 3′ RACE results of Cbln1. (B) Sequence of Cbln1. (C) Electrophoresis gel images of the 5′ and 3′ RACE results of lncRNA-PM. (D) Sequence of lncRNA-PM. All the data of this figure can be found in the S2 Data file. Cbln1, Cerebellin-1; lncRNA-PM, lncRNA-Promoting Methylation; RACE, rapid amplification of cDNA ends. (TIF) [file pbio.3001297.s002.tif]

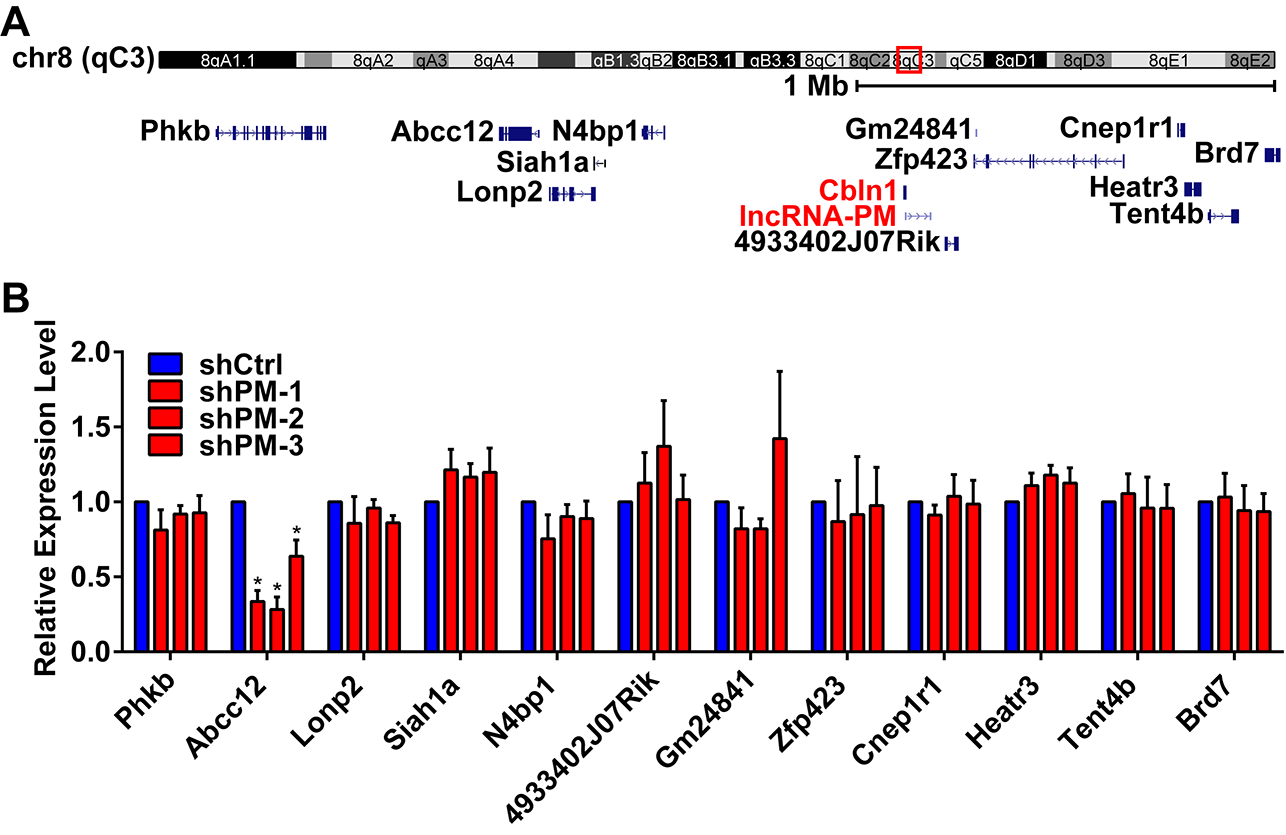

Supplement: S3 Fig — (A) Illustration of the Cbln1, Gm2694, and the tested 12 nearby genes. (B) Relative expression levels of the indicated 12 genes upon the indicated treatments in Neuro2a cells. All the data of this figure can be found in the S1 Data file. Data are shown as means ± SEMs, n = 3. *P < 0.05, **P < 0.01, and ***P < 0.001. Cbln1, Cerebellin-1; lncRNA-PM, lncRNA-Promoting Methylation. (TIF) [file pbio.3001297.s003.tif]

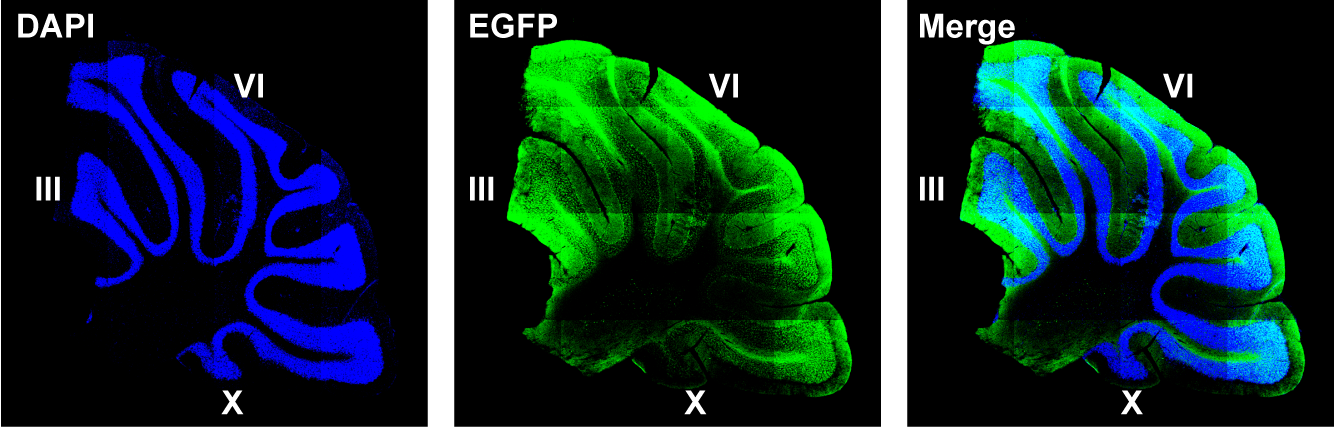

Supplement: S4 Fig — eGFP, enhanced green fluorescent protein. (TIF) [file pbio.3001297.s004.tif]

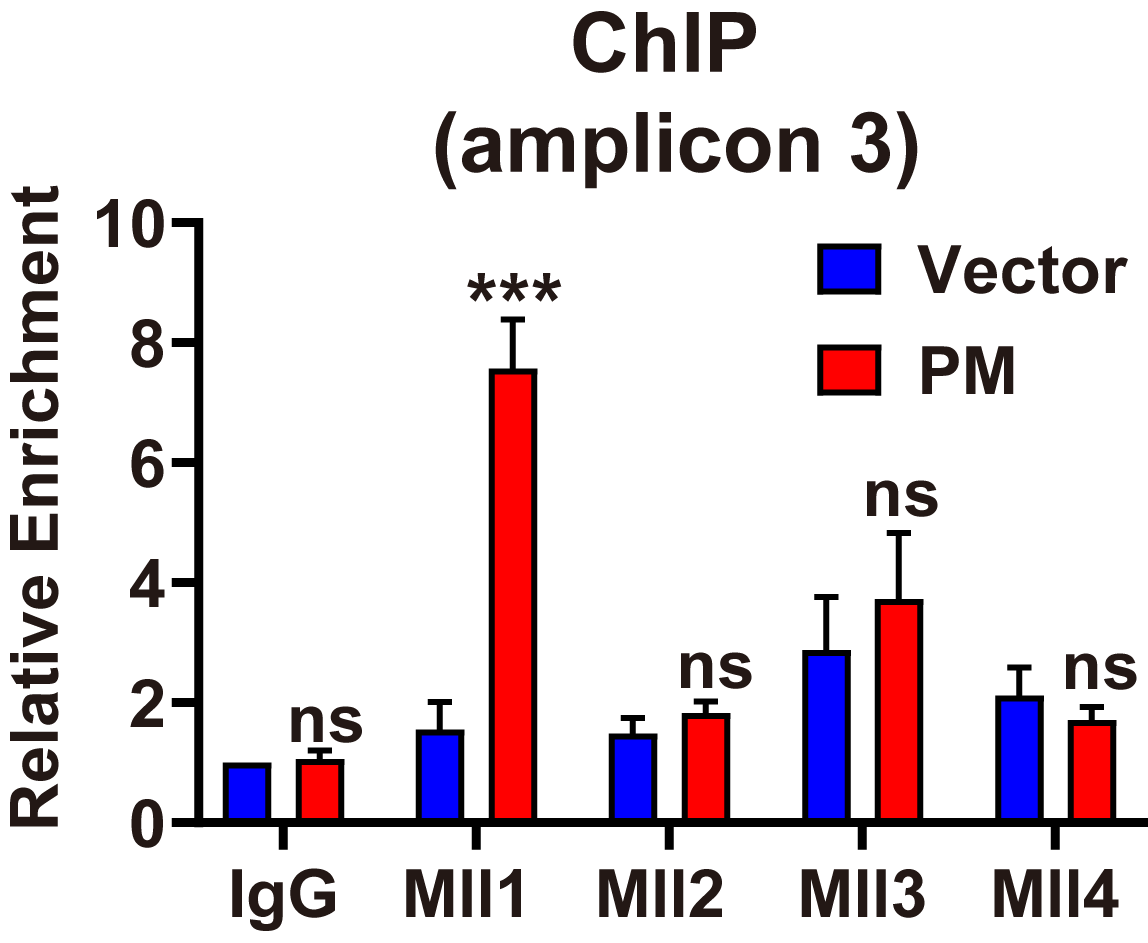

Supplement: S5 Fig — ChIP-qPCR detection of Mll1, Mll2, Mll3, and Mll4 on the indicated upstream regulatory regions of Cbln1 in the control or PM-overexpressed Neuro2a cells. All the data of this figure can be found in the S1 Data file. Data are shown as means ± SEMs, n = 3. ***P < 0.001. Cbln1, Cerebellin-1; ChIP, chromatin immunoprecipitation; IgG, immunoglobulin G; lncRNA-PM, lncRNA-Promoting Methylation; ns, no significance; qPCR, quantitative PCR. (TIF) [file pbio.3001297.s005.tif]

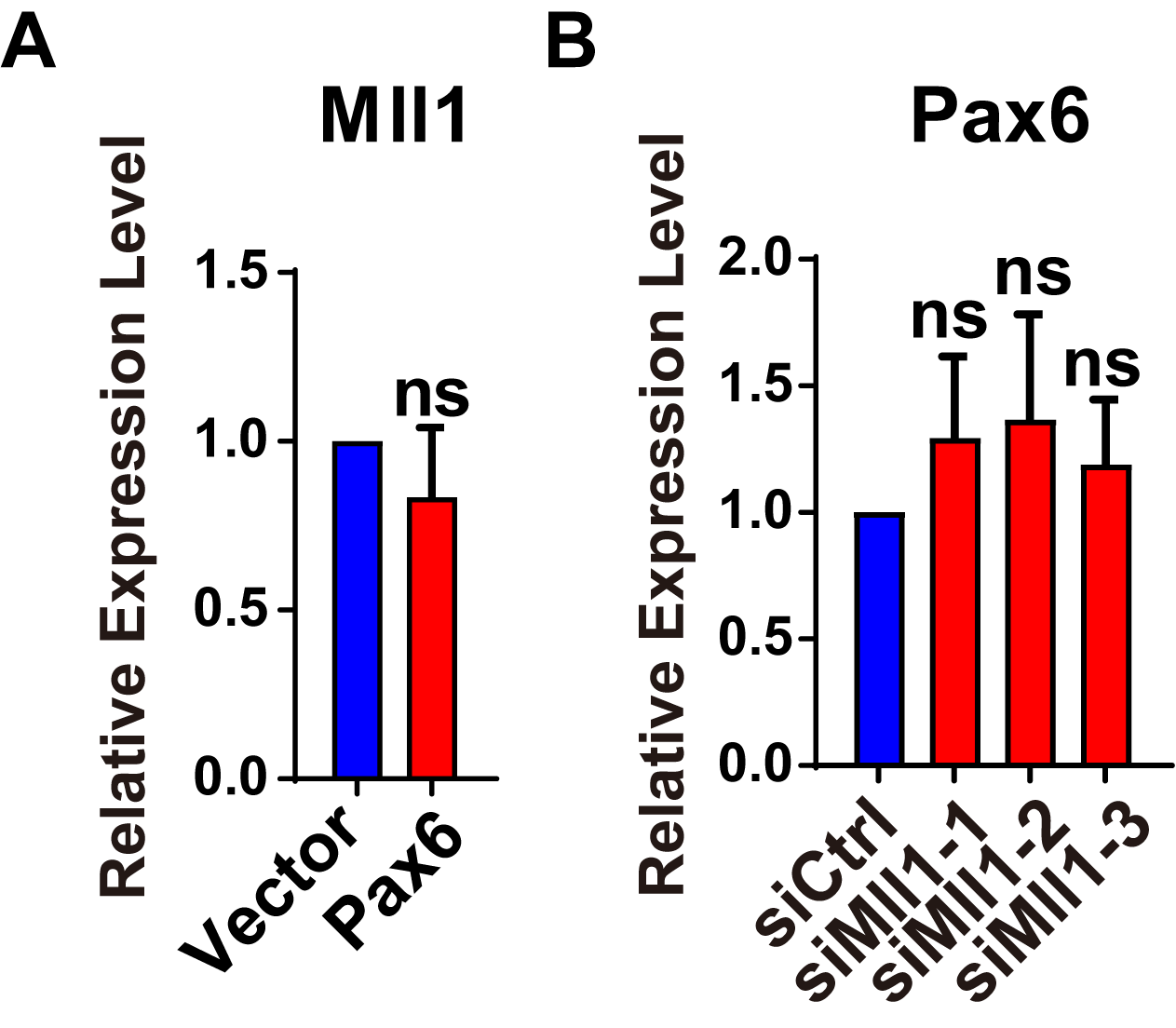

Supplement: S6 Fig — The relative expression levels of Mll1 (A) and Pax6 (B) under the indicated treatments in Neuro2a cells. All results were normalized to Gapdh. All the data of this figure can be found in the S1 Data file. Data are shown as means ± SEMs, n = 3. ns, no significance. (TIF) [file pbio.3001297.s006.tif]

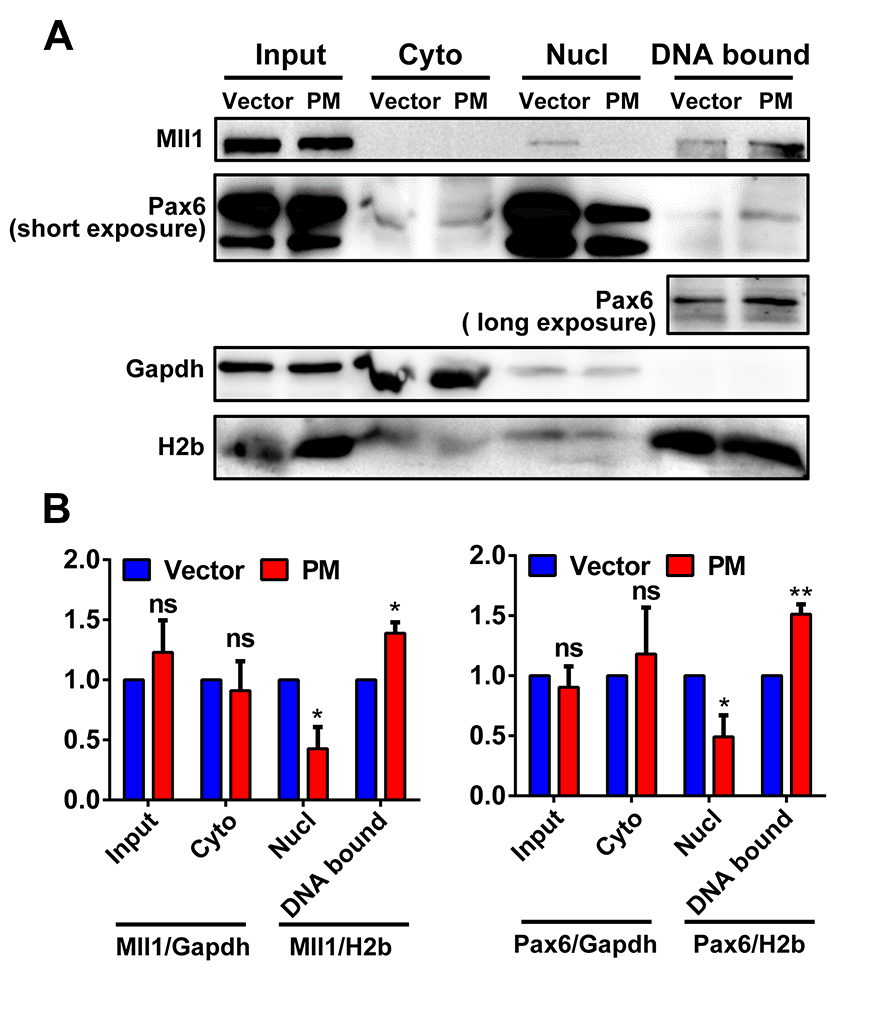

Supplement: S7 Fig — (A) Representative images of the effect of lncRNA-PM on the protein levels of Pax6 and Mll1 in the whole cell lysates (Input), cytoplasm (Cyto), nucleoplasm (Nucl), and DNA-bound fractions. (B) Quantification of A. Gapdh and H2b are markers for cytoplasm and nucleus fractions, respectively. All the data of this figure can be found in the S1 and S2 Data files. Data are shown as means ± SEMs, n = 3. *P < 0.05 and **P < 0.01. lncRNA-PM, lncRNA-Promoting Methylation; ns, no significance. (TIF) [file pbio.3001297.s007.tif]

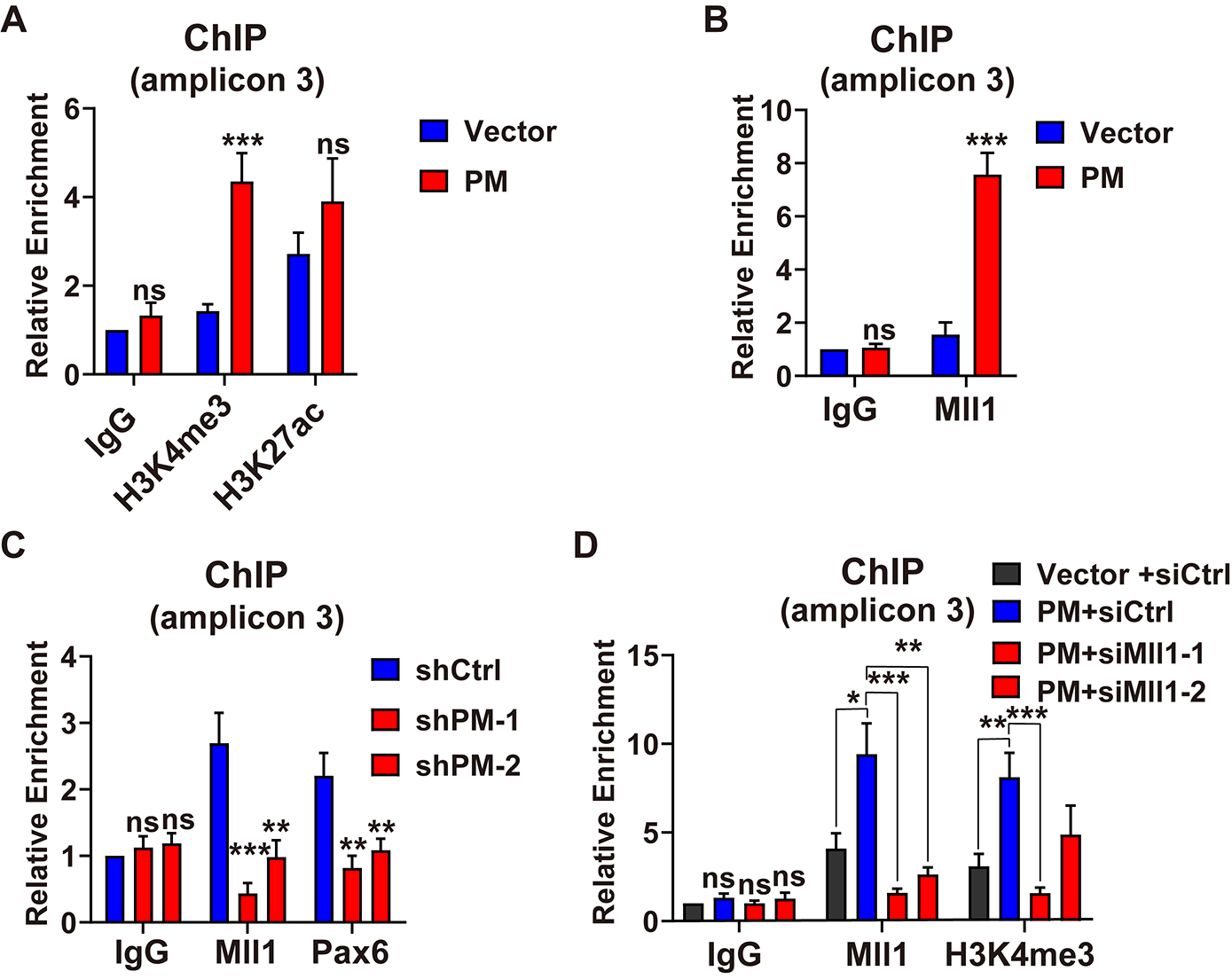

Supplement: S8 Fig — (A, B) Occupancies of H3K4me3 and H3K27ac (A) and Mll1 (B) on the 5′ regulatory region of Cbln1 detected by amplicon 3 (indicated in Fig 6C) in the control (Vector) or PM-overexpressed (PM) Neuro2a cells. Data are shown as means ± SEMs, n = 3. (C) Occupancies of Mll1 and Pax6 on the 5′ regulatory region of Cbln1 detected by amplicon 3 (indicated in Fig 6C) in the control or the indicated shPMs in Neuro2a cells. Data are shown as means ± SEMs, n = 3. (D) Occupancies of the Mll1 and H3K4me3 on the 5′ regulatory region of Cbln1 detected by amplicon 3 (indicated in Fig 6C) in PM-overexpressed Neuro2a cells, with the treatments of Mll1 (siMll1-1 and siMll1-2) or control siRNAs (siCtrl). All the data of this figure can be found in the S1 Data file. Data are shown as means ± SEMs, n = 3. *P < 0.05, **P < 0.01, and ***P < 0.001. Cbln1, Cerebellin-1; ChIP, chromatin immunoprecipitation; IgG, immunoglobulin G; lncRNA-PM, lncRNA-Promoting Methylation; ns, no significance. (TIF) [file pbio.3001297.s008.tif]

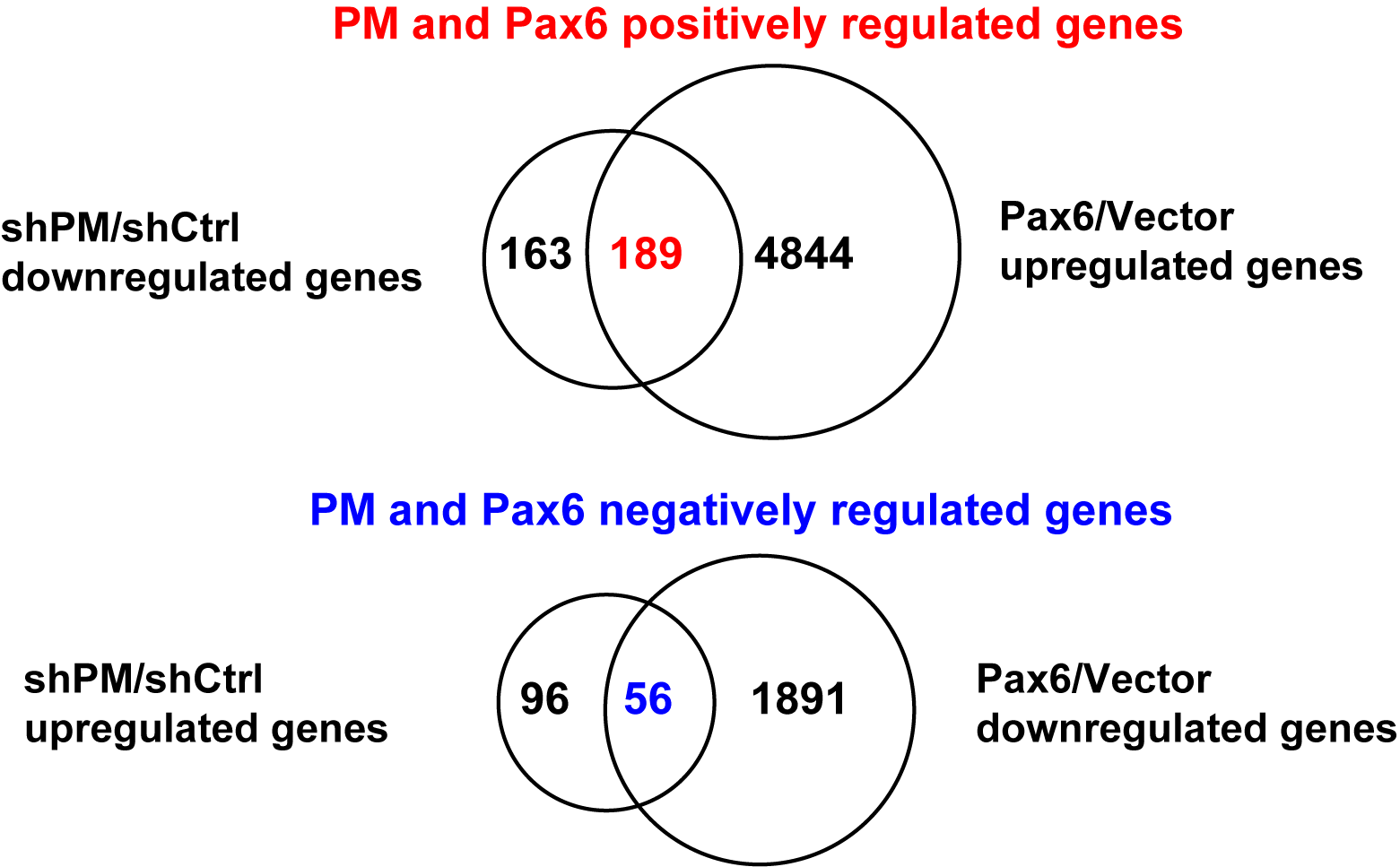

Supplement: S9 Fig — (A) Venn diagram of the positively regulated genes shared by PM and Pax6. (B) Venn diagram of the negatively regulated genes shared by PM and Pax6. lncRNA-PM, lncRNA-Promoting Methylation. (TIF) [file pbio.3001297.s009.tif]

**Figure 6B**

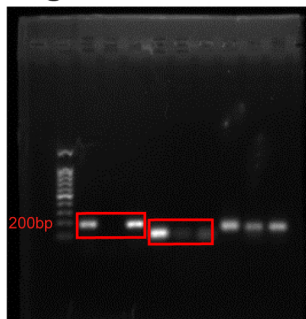

**Figure S2A**

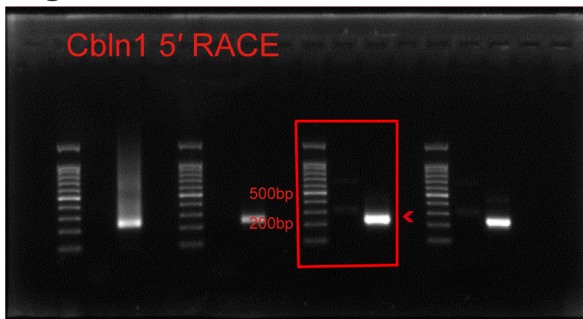

**Figure S2C**

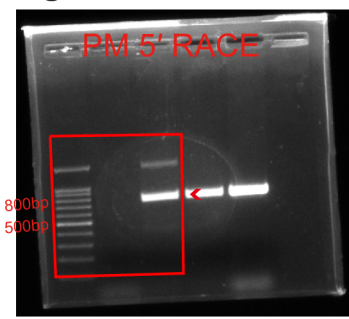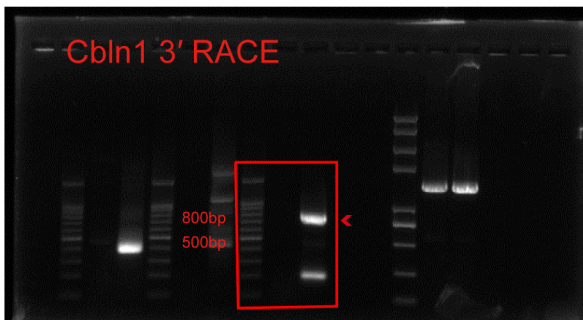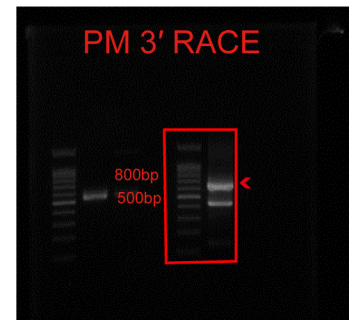

**Figure S7A**

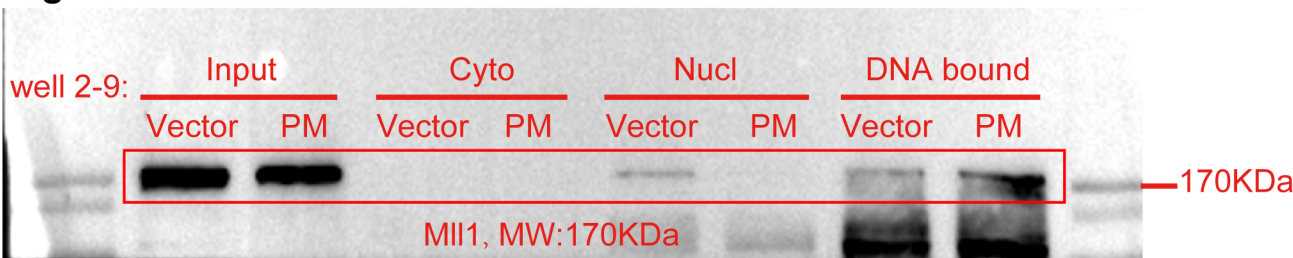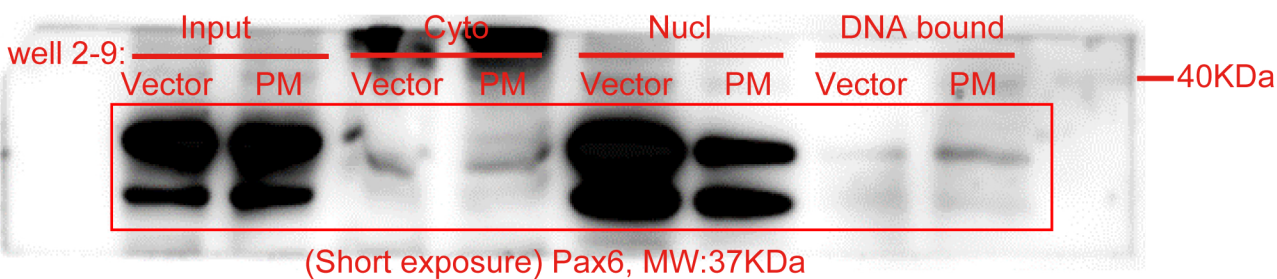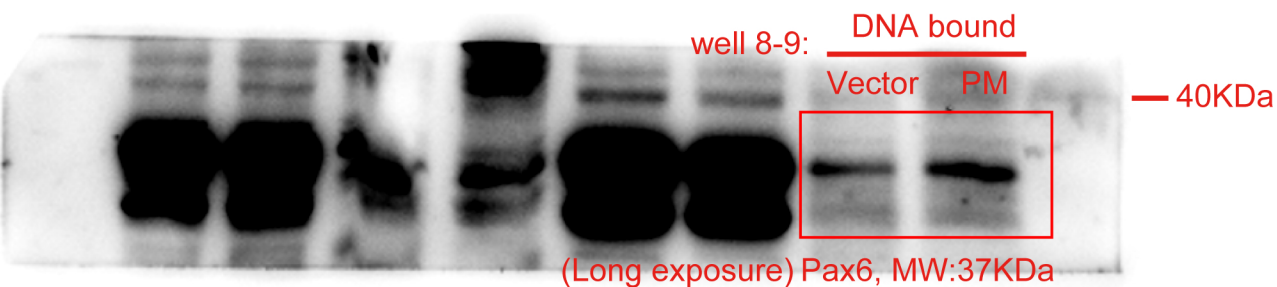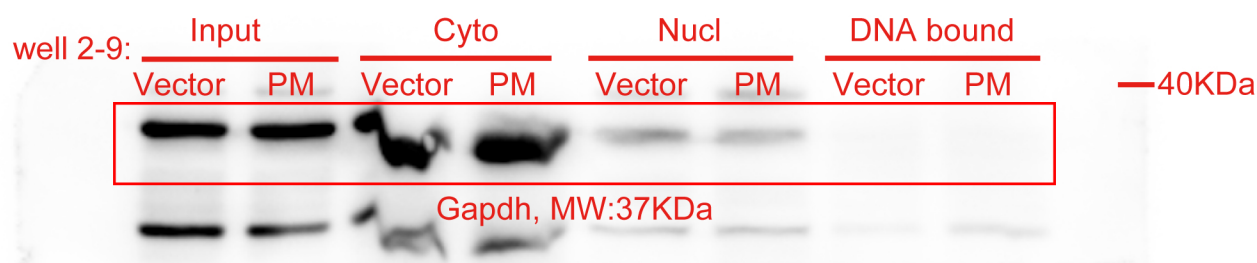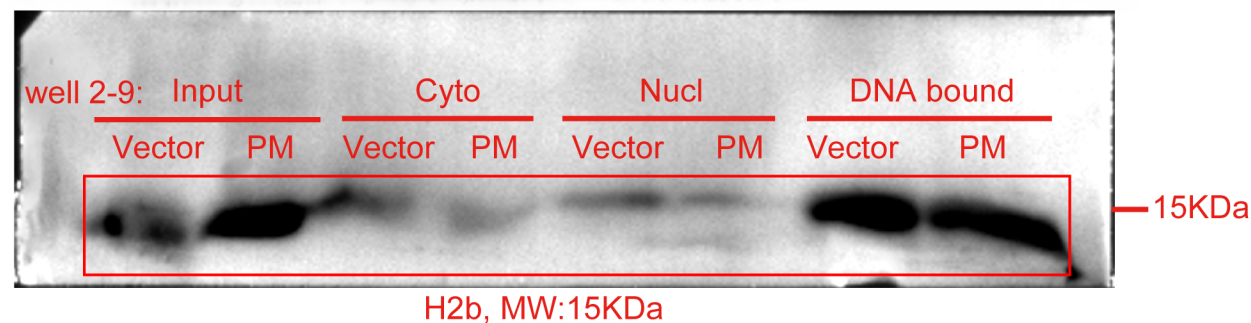

Supplement: S2 Data — (PDF) [file pbio.3001297.s018.pdf]
