# Supplementary material for: Long noncoding RNA PM maintains cerebellar synaptic integrity and Cbln1 activation via Pax6/Mll1-mediated H3K4me3
Source: PLoS Biol. 2021 Jun 10;19(6):e3001297. doi: 10.1371/journal.pbio.3001297 (PMC8219131; doi:10.1371/journal.pbio.3001297)
Supplement: S1 Table — (DOCX) [file pbio.3001297.s010.docx]

**S1 Table. List of 57 lncRNAs that are abundantly**

**expressed in the cerebellum**

| **ID** | **Gene ID** | **Chromosomal localization** | **Types** | **Neighbouring gene** |
| --- | --- | --- | --- | --- |
| 1 | 2310015B20Rik | chr10 | intergenic | / |
| 2 | 2610035F20Rik | chr14 | intergenic | / |
| 3 | 4930594M22Rik^*^ | chr14 | bidirectional | Ggact |
| 4 | 5430421F17Rik^*^ | chr8 | intergenic | / |
| 5 | 5830416P10Rik^*^ | chr19 | intergenic | / |
| 6 | 5930438M14Rik^*^ | chr13 | intergenic | / |
| 7 | Adamts18 | chr8 | exon sense-overlapping | Adamts18 |
| 8 | AI118078^*^ | chr9 | exon sense-overlapping | AI118078 |
| 9 | AK005214 | chr10 | exon sense-overlapping | Atoh7 |
| 10 | AK013470^*^ | chr19 | intergenic | / |
| 11 | AK035124 | chr7 | intergenic | / |
| 12 | AK035765^*^ | chr5 | intergenic | / |
| 13 | AK036371 | chr2 | intergenic | / |
| 14 | AK042753 | chr9 | bidirectional | Zic4 |
| 15 | AK042933^*^ | chr5 | intergenic | / |
| 16 | AK047300^*^ | chr6 | intergenic | / |
| 17 | AK047461^*^ | chr4 | intergenic | / |
| 18 | AK049103^*^ | chr14 | intergenic | / |
| 19 | AK085671^*^ | chr4 | intergenic | / |
| 20 | AK140673^*^ | chr5 | intergenic | / |
| 21 | AK144508^*^ | chr6 | bidirectional | Wnt7a |
| 22 | AK146979 | chr13 | intergenic | / |
| 23 | AK157480^*^ | chr1 | intergenic | / |
| 24 | BB365896^*^ | chr6 | intronic antisense | Fam13a |
| 25 | Chd7^*^ | chr4 | exon sense-overlapping | Chd7 |
| 26 | Chn2 | chr6 | exon sense-overlapping | Chn2 |
| 27 | Dnase2a^*^ | chr8 | natural antisense | Mast1 |
| 28 | Exph5 | chr9 | exon sense-overlapping | Exph5 |
| 29 | Gm12116^*^ | chr11 | natural antisense | Ranbp17 |
| 30 | Gm12542^*^ | chr4 | intronic antisense | Snx30 |
| 31 | Gm12985^*^ | chr4 | intron sense-overlapping | Rps6ka1 |
| 32 | Gm13828^*^ | chr5 | intergenic | / |
|  | | | | |
| **Continued** | | | | |
| 33 | Gm13944^*^ | chr2 | intronic antisense | Zfp385b |
| 34 | Gm15179 | chr1 | bidirectional | Speg |
| 35 | Gm15472^*^ | chr3 | intron sense-overlapping | Ptpn22 |
| 36 | Gm15577^*^ | chr3 | intronic antisense | Negr1 |
| 37 | Gm20743^*^ | chr1 | intergenic | / |
| 38 | Gm26686^*^ | chr1 | intergenic | / |
| 39 | Gm26755^*^ | chr2 | natural antisense | Neurod1 |
| 40 | Gm2694^*^ | chr8 | bidirectional | Cbln1 |
| 41 | humanlincRNA2001^*^ | chr5 | intergenic | / |
| 42 | Il16 | chr7 | exon sense-overlapping | Il16 |
| 43 | Iltifb | chr10 | exon sense-overlapping | Iltifb |
| 44 | Lhx1os^*^ | chr11 | bidirectional | Lhx1 |
| 45 | Mapk12 | chr15 | exon sense-overlapping | Mapk12 |
| 46 | Pld5^*^ | chr1 | exon sense-overlapping | Pld5 |
| 47 | Plekhd1os^*^ | chr12 | bidirectional | Plekhd1 |
| 48 | Rgs8^*^ | chr1 | exon sense-overlapping | Rgs8 |
| 49 | Rnf122^*^ | chr8 | exon sense-overlapping | Rnf122 |
| 50 | Sycp1-ps1 | chr7 | natural antisense | Psg19 |
| 51 | Terc | chr3 | intergenic | / |
| 52 | uc.422 | chr18 | intron sense-overlapping | Zfp521 |
| 53 | uc.423 | chr18 | intron sense-overlapping | Zfp521 |
| 54 | XLOC_000711 | chr1 | intergenic | / |
| 55 | XLOC_006584 | chr13 | intergenic | / |
| 56 | XLOC_008880 | chr15 | intergenic | / |
| 57 | Xpo4 | chr14 | exon sense-overlapping | Xpo4 |

*，validated by RT-qPCR
