# Supplementary material for: Long noncoding RNA PM maintains cerebellar synaptic integrity and Cbln1 activation via Pax6/Mll1-mediated H3K4me3
Source: PLoS Biol. 2021 Jun 10;19(6):e3001297. doi: 10.1371/journal.pbio.3001297 (PMC8219131; doi:10.1371/journal.pbio.3001297)
Supplement: S2 Table — (DOCX) [file pbio.3001297.s011.docx]

**S2 Table. List of ChIP-seq Data Files**

| **Target** | **File type** | **Accession ID** | **Biosample summary** |
| --- | --- | --- | --- |
| H3K4me1 | bam | ENCFF693CWR | H3K4me1 ChIP-seq on 8-week mouse cerebellum |
|  |  | ENCFF414ERP |  |
| H3K4me3 | bam | ENCFF087DDW | H3K4me3 ChIP-seq on 8-week mouse cerebellum |
|  |  | ENCFF693UTV |  |
| H3K27ac | bam | ENCFF461JKM | H3K27ac ChIP-seq on 8-week mouse cerebellum |
|  |  | ENCFF987PDS |  |
| Control | bam | ENCFF389ARL | Control ChIP-seq on 8-week mouse cerebellum |
|  |  | ENCFF111GKZ |  |

All files were downloaded from ENCODE.
