# Supplementary material for: Long noncoding RNA PM maintains cerebellar synaptic integrity and Cbln1 activation via Pax6/Mll1-mediated H3K4me3
Source: PLoS Biol. 2021 Jun 10;19(6):e3001297. doi: 10.1371/journal.pbio.3001297 (PMC8219131; doi:10.1371/journal.pbio.3001297)
Supplement: S4 Table — (DOCX) [file pbio.3001297.s013.docx]

**S4 Table. List of shRNAs and siRNAs**

| **Name** | **Sequences** |
| --- | --- |
| shPM-1 | ACCCGAGATTGACAAAGTG |
| shPM-2 | ACAATACTAACACTAGAGC |
| shPM-3 | ATCCCAAGAACCAAGAAGG |
| shPax6-1 | AAAGGAATGATACAAACTTGG |
| shPax6-2 | AAATGAGTCCTGTTGAAGTGG |
| shPax6-3 | TAGTTTATCATACATGCCGTC |
| siMll1-1 | GAGCCATTCTCGTCAAATA |
| siMll1-2 | GGATCAGAGTGGACTTTAA |
| siMll1-3 | GAACAAGGTTTGATATTCA |
