# Supplementary material for: Long noncoding RNA PM maintains cerebellar synaptic integrity and Cbln1 activation via Pax6/Mll1-mediated H3K4me3
Source: PLoS Biol. 2021 Jun 10;19(6):e3001297. doi: 10.1371/journal.pbio.3001297 (PMC8219131; doi:10.1371/journal.pbio.3001297)
Supplement: S5 Table — (DOCX) [file pbio.3001297.s014.docx]

**S5 Table. List of RT-qPCR primers**

| **Name** | **Sequences** |
| --- | --- |
| Gapdh-F | AGGTCGGTGTGAACGGATTTG |
| Gapdh-R | TGTAGACCATGTAGTTGAGGTCA |
| Pax6-F | CAGAGAAGACAGGCCAGCAA |
| Pax6-R | GTTTGCCATGGTGAAGCTGG |
| Cbln1-F | GAGCCTCATGTTGAACGGGT |
| Cbln1-R | GTTGAGTACTTCCAGCCCCC |
| Mll1-F | TCTCCTCTGATGCTCTGGCT |
| Mll1-R | TTTGATCGTGCCTCCTGGAC |
| Gm2694-204/PM-F | GCTGCGTTAGAACAACGGTC |
| Gm2694-204/PM-R | TGATGGGAAACCGTGAGTGG |
| Gm2694-201-F | TTCGTGGGCTTTCAGTTACCA |
| Gm2694-201-R | AAATGCCAAGGGCTAACAGGA |
| Gm2694-202-F | CGCCCGAGCCTTCTACTTG |
| Gm2694-202-R | CGGGAGTAGATCTGACCAACC |
| Gm2694-203-F | CAGCTGAACACAGGACCAGA |
| Gm2694-203-R | GCAAGAAGAATCTGGCGATGC |
| Gm2694-205-F | TGGGCTTTCAGTTACCAAGCA |
| Gm2694-205-R | GCATGCGGCTGTGTTCAG |
| Gm2694-206-F | AGACATCCAAACGACCGGGA |
| Gm2694-206-R | TAGGGCTGCCTCCAGAAATAC |
| Gm2694-207-F | AAGACATCCAAACGACCGGG |
| Gm2694-207-R | GCAGGGTGGGTATCCTGTTT |
| Gm2694-208-F | CCTCCCAACTTCCGCTTCTAT |
| Gm2694-208-R | TGGAGACGTTAGGCTGAGAGTA |
| Gm2694-209-F | TGCCTTGCCATCCAAGAGAG |
| Gm2694-209-R | CCGGGATGGTACTGGAAACC |
| Gm2694-210-F | GTGAAACTACATCCACGCCC |
| Gm2694-210-R | GGGCTGCCTCCAGAAATACG |
| Gm2694-211-F | TCCAAACGACCGGGAGTCA |
| Gm2694-211-R | CAGAGTGGGAACGGGAGTAG |
| Gm2694-212-F | GAAACAACTGCCCTTCTCCG |
| Gm2694-212-R | GGAATGGACACACTCGGACAT |
| Gm2694-F | GAAACAACTGCCCTTCTCCG |
|  | |
| **Continued** | |
| Gm2694-R | GGGGACAGTGAGCAAGGAAA |
| 4933402J07Rik-F | AAACAAGGCTCCCGACAACA |
| 4933402J07Rik-R | TTTGCGTCTATCTCGGCCTC |
| Zfp423-F | TTGCCTTTTGGGCACCCTG |
| Zfp423-R | CTTCAAGTAGCTCAGGCGGAT |
| Gm24841-F | AATGCAAAGGTATTGGGTGAGTTTT |
| Gm24841-R | TGATCTCCACAGCCAGCTTTA |
| Cnep1r1-F | GTAGTCGCACCATCAATTATAGC |
| Cnep1r1-R | TCTCTAGCAACAGTGCGGA |
| Heatr3-F | TGAAACCCAGCGGCTAAAGA |
| Heatr3-R | GCTCCTTGTCAGTAGGTGGAA |
| Tent4b-F | GAGTCATGCAGTGTCACCGA |
| Tent4b-R | ATTCTGCAAGCCCCACTGTT |
| Brd7-F | GACCGCCACTTCTACGAGG |
| Brd7-R | TGTTTGTCATGGTCGCTTCTG |
| N4bp1-F | AAAACGAACCAGGACGAGCA |
| N4bp1-R | ACTTTTTCAGACCGTGGGTAA |
| Siah1a-F | AAGGTAGCAGAGCAGCAGTC |
| Siah1a-R | TTGCCCCAGACATTTGAAGAGA |
| Lonp2-F | GTTCGGATGTGGCTGATGGA |
| Lonp2-R | CGGTCAGTGTTAGTTGGCCT |
| Abcc12-F | TGTACGCCTGGGAGGAATCT |
| Abcc12-R | AGTGAGCTTGCGTTTCAGGA |
| Phkb-F | TCCAACTACCGGTCTCTTTCC |
| Phkb-R | GGTCCGTCCCTTGTCATCAT |
| Dlx2-F | TCAACAACGAGCCGGACAAG |
| Dlx2-R | CTGGAGTAGATGGTGCGTGG |
| Csf1-F | AAAGTTTGCCTCGGTGCTCT |
| Csf1-R | GCCCAGCCATGTCGAAGAA |
| Cnr2-F | CCTGTTGCTGTGTGCATCCT |
| Cnr2-R | ATGGATGGGCTTTGGCTTCT |
| Jak2-F | CTGCACAAAACGGAAGTTCTTT |
| Jak2-R | GCTTAGCCACTCCAAGTTTCC |
| Bbc3-F | GTGGATCTGCAGGTGTCTCG |
|  | |
| **Continued** | |
| Bbc3-R | GGGCTAGACCCTCTACGGG |
| Btg2-F | GCCAGACCGTCATCATCGT |
| Btg2-R | ACCAGTGGTGTTTGTAATGATCGGT |
| Ddit3-F | TCTTGAGCCTAACACGTCGATT |
| Ddit3-R | ACGTGGACCAGGTTCTCTCT |
| Fos-F | AGAGCGGGAATGGTGAAGAC |
| Fos-R | AGTTGATCTGTCTCCGCTTGG |
| Rel-F | GGACATTGAAGACTGCGACC |
| Rel-R | GCAGTATTTGGGGCACGGTT |
| Icam1-F | AGCCTCCGGACTTTCGATCT |
| Icam1-R | TGTTTGTGCTCTCCTGGGTC |
| Plcl1-F | GTGCCATTCCAGATGTGCAG |
| Plcl1-R | CAGGTCTCCTTGTCCCTTCAAT |
| Egr2-F | CGGATCACAGGCAGGAGAGA |
| Egr2-R | TCGGATACGGGAGATCCAGG |
| Tbx3-F | CTACGGGGGAGCAATGGATG |
| Tbx3-R | GGGAAGGCCAAAGTAAATCCG |
| Grhl2-F | TCTTGTTCTGCCATCTCGGG |
| Grhl2-R | TGAACTCGCTCCCCTTTTCC |
| Nr1d1-F | GCAAGGGCACAAGCAACATT |
| Nr1d1-R | TGCACTCCATAGTGGAAGCC |
| Il6-F | CAACGATGATGCACTTGCAGA |
| Il6-R | GTGACTCCAGCTTATCTCTTGGT |
| Idh2-F | TGCCCTGATGGGAAGACAAT |
| Idh2-R | AGCGTCTGTGCAAACCTGATAA |
| Ldlr-F | TCAATGGGGGCAATCGGAAA |
| Ldlr-R | ACAGTGTCGACTTCTCTAGGC |
