# Supplementary material for: Long noncoding RNA PM maintains cerebellar synaptic integrity and Cbln1 activation via Pax6/Mll1-mediated H3K4me3
Source: PLoS Biol. 2021 Jun 10;19(6):e3001297. doi: 10.1371/journal.pbio.3001297 (PMC8219131; doi:10.1371/journal.pbio.3001297)
Supplement: S6 Table — (DOCX) [file pbio.3001297.s015.docx]

**S6 Table. List of qPCR primers for ChIP and ChIRP**

| **Name** | **Sequences** |
| --- | --- |
| Far Region-F | GAGTGCCTGCTCTCCTCATC |
| Far Region-R | CTGTCCCTGTCCATGTGTCC |
| Cbln1-1F(amplicon1) | CCTCCTCAGCTCTGTGCG |
| Cbln1-1R(amplicon1) | GCGCCCGCTGCATCAATAAT |
| Cbln1-2F(amplicon2) | CAGCCAATCGCGACGCA |
| Cbln1-2R(amplicon2) | CCTCGCCGCTCCTAATAACA |
| Cbln1-3F(amplicon3) | CGGGAGTCAGACGAGGGA |
| Cbln1-3R(amplicon3) | ACGTACGTGCCCTTAACCAG |
| Cbln1-4F(amplicon4) | GGGAAATGGAGTGGACACCG |
| Cbln1-4R(amplicon4) | GGCGCATAGCTCCTACCAG |
| Cbln1-5F(amplicon5) | TGCACTTGTCCACGGTAGATT |
| Cbln1-5R(amplicon5) | CTCCTCACCTCATCTCCCACT |
| Cbln1-6F(amplicon6) | CAGGAACGCAGACAGGTAGG |
| Cbln1-6R(amplicon6) | GAGGCTGTCTCGAGATTCGG |
| Cbln1-7F(amplicon7) | GAGACAGCCTCACCTAGCC |
| Cbln1-7R(amplicon7) | AGGCAGGCTTCAGAAAAGAGG |
| Cbln1-8F(amplicon8) | TAGAGCAAGCAGCAGTTCACA |
| Cbln1-8R(amplicon8) | CCTTATTTCATGCCAGCGCA |
| Cbln1-9F(amplicon9) | CTCATACTCATGCGCTGGCA |
| Cbln1-9R(amplicon9) | GTTAGGGCTCAGTGGGTGTT |
| Cbln1-10F(amplicon10) | GAAACACCCACTGAGCCCTA |
| Cbln1-10R(amplicon10) | TCCACCCCCACTTGTCTTGT |
| Cbln1-11F(amplicon11) | GAACAAGACAAGTGGGGGTG |
| Cbln1-11R(amplicon11) | TGGAGGCAATCTTCCCTAGACA |
| Cbln1-12F(amplicon12) | CGCCAACCTGTTCATCCTGT |
| Cbln1-12R(amplicon12) | CCTTGCAGGCCGAGAAGAAC |
| Cbln1-13F(amplicon13) | CTGTCAACCTGATGGCAGGAA |
| Cbln1-13R(amplicon13) | AAGGGCCGAGTTTCAAAAGC |
| Cbln1-14F(amplicon14) | CTTCTTTTCCTGTCCCAGCGA |
| Cbln1-14R(amplicon14) | GGCTAGCGATCTGAGCCAA |
