# Supplementary material for: Long noncoding RNA PM maintains cerebellar synaptic integrity and Cbln1 activation via Pax6/Mll1-mediated H3K4me3
Source: PLoS Biol. 2021 Jun 10;19(6):e3001297. doi: 10.1371/journal.pbio.3001297 (PMC8219131; doi:10.1371/journal.pbio.3001297)
Supplement: S7 Table — (DOCX) [file pbio.3001297.s016.docx]

**S7 Table. List of ChIRP probes**

| **Name** | **Sequences** |
| --- | --- |
| ChIRP LacZ-1 | AACGAGACGTCACGGAAAAT |
| ChIRP LacZ-2 | ATAGAGATTCGGGATTTCGG |
| ChIRP LacZ-3 | TGGTTCGGATAATGCGAACA |
| ChIRP LacZ-4 | GTTATCGCTATGACGGAACA |
| ChIRP LacZ-5 | AAATCCATTTCGCTGGTGGT |
| ChIRP LacZ-6 | TTGCCAACGCTTATTACCCA |
| ChIRP PM-1 | CTTTTGATGTTAACCGCACT |
| ChIRP PM-2 | GGAAACCGTGAGTGGTTATC |
| ChIRP PM-3 | TTGTCAGAGCAGAGCGAATC |
| ChIRP PM-4 | TGCTTAAGTGACTAGTTCCT |
| ChIRP PM-5 | TAACACTAGAGCTGAGCGGA |
| ChIRP PM-6 | TTGGATGTCTTCCCCTGAAG |
